# Serum adipokines/related inflammatory factors and ratios as predictors of infrapatellar fat pad volume in osteoarthritis: Applying comprehensive machine learning approaches

**DOI:** 10.1038/s41598-020-66330-0

**Published:** 2020-06-19

**Authors:** Hossein Bonakdari, Ginette Tardif, François Abram, Jean-Pierre Pelletier, Johanne Martel-Pelletier

**Affiliations:** 1 Osteoarthritis Research Unit, University of Montreal Hospital Research Centre (CRCHUM), Montreal, Quebec Canada; 2 Department of Soils and Agri-Food Engineering, Laval University, Quebec, Quebec, Canada; 3 Medical Imaging, ArthroLab Inc., Montreal, Quebec Canada

**Keywords:** Biomarkers, Diseases, Medical research, Pathogenesis, Risk factors, Mathematics and computing

## Abstract

Objective. The infrapatellar fat pad (IPFP) has been associated with knee osteoarthritis onset and progression. This study uses machine learning (ML) approaches to predict serum levels of some adipokines/related inflammatory factors and their ratios on knee IPFP volume of osteoarthritis patients.Methods. Serum and MRI were from the OAI at baseline. Variables comprised the 3 main osteoarthritis risk factors (age, gender, BMI), 6 adipokines, 3 inflammatory factors, and their 36 ratios. IPFP volume was assessed on MRI with a ML methodology. The best variables and models were identified in Total-cohort (n = 678), High-BMI (n = 341) and Low-BMI (n = 337), using a selection approach based on ML methods. Results. The best model for each group included three risk factors and adipsin/C-reactive protein combined for Total-cohort, adipsin/chemerin; High-BMI, chemerin/adiponectin HMW; and Low-BMI, interleukin-8. Gender separation improved the prediction (13–16%) compared to the BMI-based models. Reproducibility with osteoarthritis patients from a clinical trial was excellent (R: female 0.83, male 0.95). Pseudocodes based on gender were generated.Conclusion. This study demonstrates for the first time that the combination of the serum levels of adipokines/inflammatory factors and the three main risk factors of osteoarthritis could predict IPFP volume with high reproducibility, with the superior performance of the model accounting for gender separation.

## Introduction

Osteoarthritis (OA) is known as one of the most common musculoskeletal disorders and occurs in the about half of the population over 65 years old^1,2^. According to the World Population Data Sheet^3^, older adults (>65); comprising about 5% of the global population in 1960, increased to 9% in 2018 and are projected to rise to 16% by 2050. With this population shift, the social and economic effects of medical care related to OA will be significant; however, current treatments are only symptomatic and no cure yet exists. One of the hurdles in OA drug discovery, as well as for the improvement of therapeutic approaches, is the early identification of patients who will progress. It is therefore crucial to find efficient and reliable means of screening OA progressors. Although the main risk factors, age, gender and body mass index (BMI), are important, they alone are poor predictors. However, serum molecules could be potential biomarkers for predicting knee OA progression.

Recent studies suggest that alteration in the infrapatellar fat pad (IPFP), an adipose tissue and the largest soft tissue structure in the knee joint^4^, is a potential early marker of OA disease incidence or progression^5–9^. Some inflammatory factors and adipokines produced by the IPFP have been identified as having a role in OA progression^10,11^. Furthermore, ratios of adipokines/inflammatory factors have also been demonstrated in OA and other diseases to provide a better prediction assessment than individual factors^12–19^.

In a first step toward finding early reliable predictors of OA progressors, this study aimed to determine, in OA individuals, the optimum combination of serum levels of adipokines, related inflammatory factors, their ratios, and the three main OA risk factors for predicting knee OA IPFP volume. Moreover, as adipokines are adipose tissue-derived mediators and some are involved in obesity, we also investigated if differences occurred between obese and non-obese OA subjects. To this end, we used a comprehensive machine learning approach which provides an excellent means for such predictive assessment.

## Patients and Methods

### Study population

The Osteoarthritis Initiative (OAI) is a longitudinal, multi-center study of knee OA of 4,796 individuals aged 45–79. The target knee of the participants (details in Supplementary Materials) were selected from the Progression subcohort of the OAI database (https://oai.nih.gov) and magnetic resonance imaging (MRI) were obtained from the baseline time point of the OAI cohort as previously described^12^. The knees of 678 participants were selected for this study and participant characteristics are described in Supplementary Table S1a. Participants were further divided according to their BMI: obese (BMI ≥ 30 kg/m^2^, High (H)-BMI; n = 341) and non-obese (BMI < 30 kg/m^2^, Low (L)-BMI; n = 337) and further by gender (female, n = 290, male n = 388).

### Serum samples and biomarker determination

The serum samples were obtained from the baseline time point of the OAI cohort as previously described^12^. Serum samples were received aliquoted and frozen. Upon reception, they were stored at −80 °C and thawed to 4 °C before use. In brief, morning blood specimens were collected after an overnight fast using a uniform protocol. Additional details on specimen collection and processing methods can be found in the OAI operations manuals (https://oai.nih.gov).

The markers included the three main OA risk factors (age, gender and BMI), 9 serum biomarkers (6 adipokines: adiponectin high [H] and low [L] molecular weight [MW], adipsin, chemerin, leptin, visfatin, and three related inflammatory factors: C-reactive protein [CRP], interleukin [IL]-8, monocyte chemoattractant protein-1 [MCP-1]), and their 36 ratios. All biomarkers were determined with specific assays according to the manufacturers’ specifications as previously described (for details about the methodology for Biomarker determination, refer to Supplementary Materials).

All OAI participants provided written informed consent for participation in the OAI. Ethics approval was obtained by each OAI clinical site (University of Maryland Baltimore—Institutional Review Board, Ohio State University’s Biomedical Sciences Institutional Review Board, University of Pittsburgh Institutional Review Board, and Memorial Hospital of Rhode Island Institutional Review Board) and the OAI coordinating center (Committee on Human Research at University of California, San Francisco, CA, USA).

The Institutional Ethics Committee Board of the University of Montreal Hospital Research Centre approved the use of the human serum.

All methods, including serum measurements, were performed in accordance with the relevant guidelines and regulations.

### Infrapatellar fat pad (IPFP) volume assessment using MRI

Knee MRI acquisitions were performed at the four OAI imaging centers using a 3 T apparatus (Magnetom Trio, Siemens, Erlangen, Germany). The MR coronal intermediate weighted (COR IW) 2D TSE sequence as defined by the OAI protocol^20^ was used for segmentation. The automated IPFP segmentation was done using a convolutional neural network (CNN). In brief, it implements a multi-atlas segmentation^21^ approach based on a multi-label pixel wise CNN^22^ trained on 2 labels: the first representing the IPFP and the second the knee joint bones including femur, tibia, patella and fibula as a single class. The core of the technology is a U-net architecture implemented in the MxNet framework which includes 5 convolution + pooling layers followed by 5 deconvolution + pooling layers^23^. The IPFP volume is expressed in mm^3^. Validation done on 38 individuals (Supplementary Table S1b) and comparing the manual segmentation with the developed CNN methodology revealed an excellent correlation coefficient, R = 0.90. Detailed methods are in Supplementary Materials.

### Determination of the best variable combinations for IPFP determination

As a first step, the correlation coefficient (normalized version of the covariance) values are performed in order to measure the significance between the factors under study. Although the values of such a measure show both the tendency and strength of the linear relationship among two variables, when there are more than two interventions, the interaction between all variables should be evaluated with a more appropriate method. The latter should take into account the relationship and interactions between two or more factors. Therefore, and in order to consider all possible combinations between the factors under study on IPFP volume, we further used an evolutionary-based variable selection method for selecting the best variable combinations in IPFP volume prediction from 48 variables. The best variable combinations were determined using the evolutionary algorithm particle swarm optimization (PSO), which is combined with an artificial neural network (ANN) and Monte-Carlo simulation (MCS).

Although artificial intelligence techniques have been developed to address different practical needs in different areas, each of these methods has different pros and cons. For this study, 2.81E + 14 sub-variables should be analyzed to identify the best ones (Supplementary Table S2). Therefore, in feature selection, ANN was chosen as it could address our main goal, which was to develop an easy approach for implementation via the ability of classification, and pattern recognition (detailed method in Supplementary Materials). Moreover, as the dataset may possess some redundant or irrelevant features, the extraction of coherent information requires a comprehensive search over the sample space, while evolutionary algorithms accelerate the learning process to solve the problem. In order to find the best variable among models for IPFP volume determination with the sub-variables, a PSO-based variable selection algorithm was encoded as such technology has been successfully applied to various areas including feature selection^24–27^. A PSO algorithm was employed to select the most effective features as it can balance exploration and exploitation in an optimal manner by combining local and global search methods through self and neighboring experiences^28^. Other advantages of PSO that were needed in the present study are the fast search speed, simple algorithm structure with few adjustable parameters, and possessing memory.

To find the optimal variables (dimensional [12 dimensional: three risk factors and the serum levels of 9 adipokines/inflammatory factors], ratios, or both) for IPFP volume prediction models, PSO-based variable selection method was used and was calculated as follows:

1 $${C}_{M}^{N}=\frac{N!}{M!(N-M)!}$$

where *C* is the number of possible combinations, *N* is the number of desired variables and *M* is the number of all sub-variables. According to the above equation, the number of sub-variables of 1 to 48 variables, as in the Supplementary Table S2, is 2.81E + 14 sub-variables that should be analyzed to identify the best ones.

For each group, 70% of the values were randomly assigned for use in training and the remaining (30%) for testing. k-fold cross-validation was employed to detect over-fitting and to verify the generalizability of the proposed models. In k-fold cross-validation, all samples were randomly divided into k categories. In each modeling period, k-1 categories were deemed training samples and the remaining sub-data were used as the testing samples. This process was repeated k times. Therefore, all samples were applied at least once as test samples. The testing samples were randomly selected from all samples. Of note, the testing dataset had no role in modeling development and is completely different from the training data. Here, by considering k = 4, the data set was split into 4 folds.

Considering all input combinations, a PSO-based variable selection method was developed (detailed methods in Supplementary Materials) to identify the most important sub-variables through proposed variable selection approach; Supplementary Fig. S1 illustrates the flow chart of the PSO-based variable selection method. In addition, the Monte Carlo Selection is employed to overcome the uncertainty in modeling results. Forty-eight (48) models were then selected.

Next, the adaptive neuro-fuzzy inference system embedded with fuzzy c-means clustering (ANFIS-FCM)^29^ was employed to predict IPFP volume. ANFIS is a multi-layer feedforward system that uses neural network learning algorithms and fuzzy logic to map an input space onto an output space (detailed method in Supplementary Materials and flowchart in Supplementary Fig. S2). Accordingly, all 48 variables were called, along with IPFP volume as the target variable, and all samples were divided into two categories of training (70% of the database) and testing (remaining 30%). The number of variables was then determined, and as in Supplementary Fig. S3 can vary from 1 to 48. After determining the variation range of the number of desired variables, and in order to evaluate the model performance in different stages, the weight of the training and testing errors was determined. This weight is proportional to the intended percentage for each stage (i.e. training and testing).

### Uncertainty analysis

The uncertainty of the ANFIS-FCM prediction of the desired variables was applied as described^30^ and included the standard deviation of the forecasting error (SDFE) and the 95% confidence band width of uncertainty band (WUB) (detailed formulas in Supplementary Materials). Although it is well-known that performing these analyses might benefit the established ANFIS-FCM model, it will also make a reasonable comparison to expose the prediction vulnerabilities of the different models when compared to actual data.

### Performance evaluation criteria

The different statistical indices for validating the performance of the developed ANFIS-FCM in IPFP volume prediction were the scatter index (SI), correlation coefficient (R), mean absolute percentage error (MAPE), and root mean squared relative error (RMSRE) (detailed formulas in Supplementary Materials).

### Reproducibility of the proposed model

Reproducibility of the ANFIS-FCM code was done using another cohort; patients with symptomatic knee OA selected from a multicenter, randomized, double-blind study evaluating the effect of Licofelone (a lipoxygenase/cyclooxygenase inhibitor) versus Naproxen (a cyclooxygenase 2 inhibitor)^31^. Eighty (80) OA patients (female, n = 57; male, n = 23) were used. Patients’ characteristics are described in the Supplementary Table S1a. The original study was approved by the respective local ethics committees and all patients gave their oral and written informed consent to participate, including permission for the use of serum to be collected for biomarker assessment. Samples from this cohort were used only for validation and had no role in the modeling development.

## Results

### Selecting the best sub-variables combination based on PSO-based variable selection (PSOBVS)

Firstly, the correlation coefficient was performed (Supplementary Table S3) between 48 variables (defined as dimensional, the three risk factors and the serum levels of nine adipokines/inflammatory factors; and their 36 ratios) and the IPFP volume. Data indicated that the correlation coefficient between the three confounding variables (age, gender, BMI) were very low. For the other factors tested and the confounding variables, the highest correlation coefficient were between gender and IPFP volume (0.58). For the adipokines, the highest correlation coefficient were found between the adiponectin HMW and adiponectin LMW (0.58); this is not surprising as both are from the same molecule^32–34^.

Next and to find the best sub-variables in IPFP volume prediction of the 48 variables, the PSOBVS was implemented through an iterative process for the Total cohort, High-BMI and Low-BMI datasets. The results in the Supplementary Table S4 represents the models with 1 to 48 variables and were obtained by considering all sub-variables related to the parametric models with 1–48 variables (Supplementary Materials, Equation 2). For each of the three groups (Total cohort, High-BMI and Low-BMI), a total of 1,712,304 models (Supplementary Table S2) were verified. The Akaike Information Criterion (AIC) index (Supplementary Materials, Equation 5) was applied to select the best sub-variables, such that the model with the lowest AIC value was deemed the best model. According to Supplementary Table S2, the total sub-variables to be investigated and that are needed to identify the top 48 sub-variables are 2.81E + 14. Since this study investigates three groups (Total cohort, Low-BMI and High-BMI), 8.44E + 14 different sub-variables were investigated and 48 sub-variables were selected (Supplementary Table S4).

Data revealed that the best variables selected corresponded for each group of the model #5 (Supplementary Table S4) consisting of 5 variables: age, gender, BMI, adipsin/CRP for each group, combined for the Total cohort with adipsin/chemerin; High-BMI, chemerin/adiponectin HMW; and Low-BMI, IL-8.

**Figure 1:**
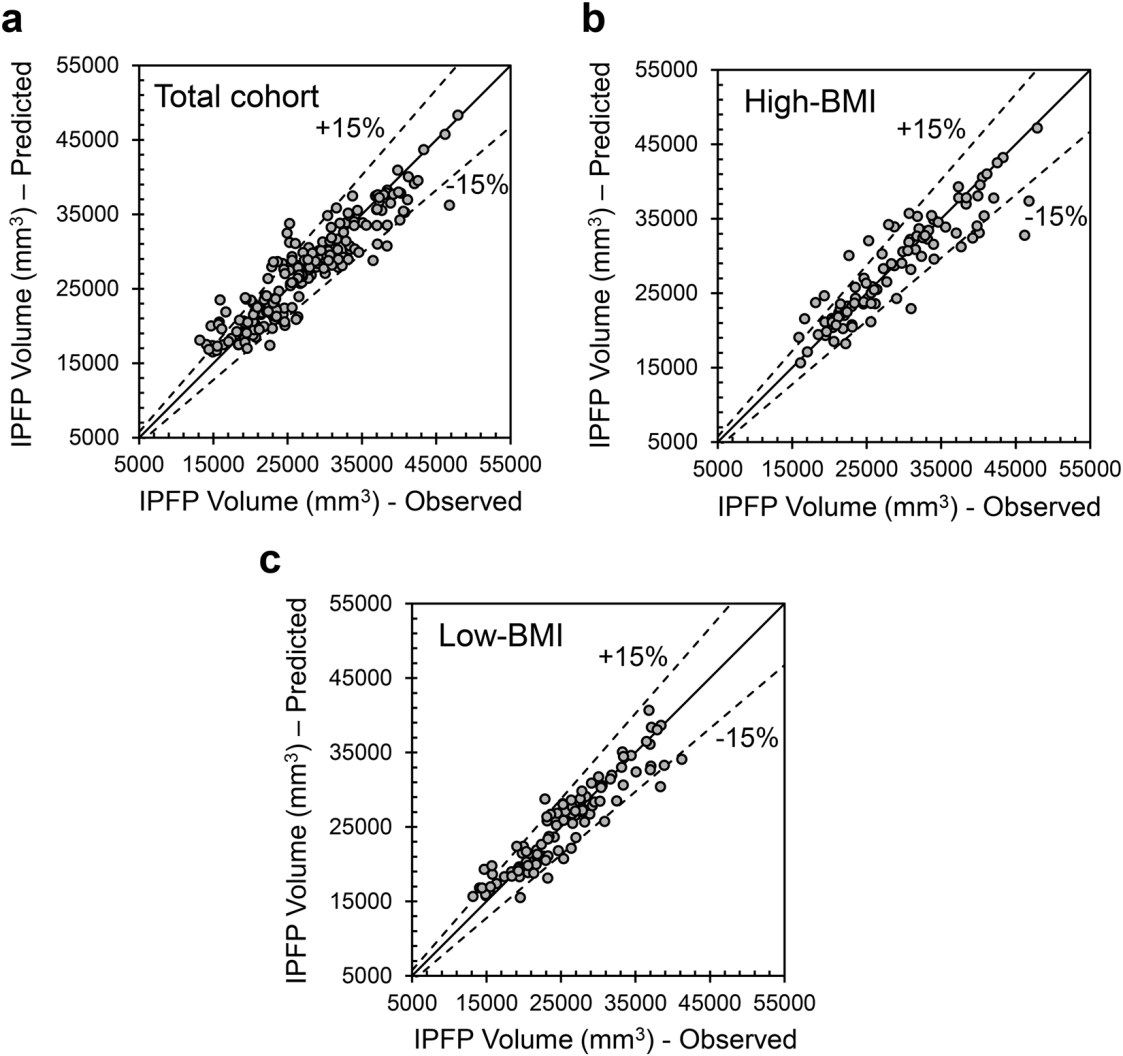
Scatter plot of infrapatellar fat pad (IPFP) volume prediction by the developed adaptive neuro-fuzzy inference system, embedded with fuzzy c-means clustering (ANFIS-FCM) in the testing stage. (**a)** Total cohort, (**b)** High-bone mass index (BMI), and (**c)** Low-BMI.

**Figure 2:**
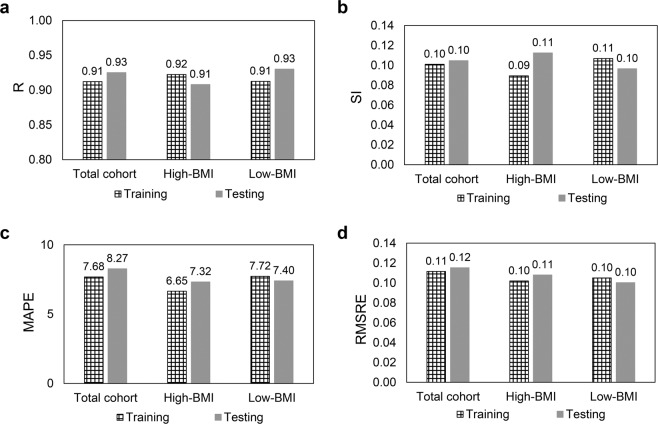
Statistical indices for the best input combinations for training and testing stages for the Total cohort, High-bone mass index (BMI), and Low-BMI. (**a)** R, correlation coefficient; (**b)** SI, scatter index; **(c)** MAPE, mean absolute percentage error; **(d)** RMSRE, root mean squared relative error.

By using ANFIS-FCM, the performance of the variables selected for each group was determined using scatter plot (Fig. 1) and statistical indices (Fig. 2). Data showed that for the Total cohort (Fig. 1a), High-BMI (Fig. 1b) and Low-BMI (Fig. 1c) in testing stage, each model estimates the IPFP volume prediction in most samples with low relative error, so that only 85% of the samples having a relative error of less than 15% and more than 75% of the samples estimate IPFP volume with relative error less than 10%. The comparison of the statistical indices (Fig. 2) for training and testing stages indicates that the developed model performs similarly in each stage, but the Total cohort shows a slightly higher performance in testing stage compared to the training one, for all the statistical indices.

### Evaluation of the inverse ratio of the selected combinations

For the validation of the selected ratios, we further investigated the performance of ANFIS-FCM in IPFP volume prediction using their inverse forms. More specifically, we examined whether the inverse of the selected ratios for the IPFP volume modeling will have an impact on the results. The statistical indices for each group studied revealed (Supplementary Fig. S4) that compared to the ratio of the main form (form used during the development), the inverse form of the ratio within each model showed, for both training and testing stages, small differences in statistical indices in favor of the main form. Accordingly, using the inverse form of the ratios rather than the main form results in slightly inferior performance, confirming the superior performance of the main form for each group.

### Impact of each variable of the main form

Next, we investigated the impact of each variable for each group on the IPFP volume prediction. To this end, 25 models with 2 to 5 sub-variables of the model #5 were evaluated for each group: Total cohort, High-BMI and Low-BMI. As illustrated in Table 1, the models (M) M1, M2-M6, M7-M15, M16-M25 correspond to 5, 4, 3 and 2 sub-variables respectively and M2-M25 were compared with the selected model with 5 variables, M1 including age, gender, BMI, adipsin/CRP in addition to the Total cohort of adipsin/chemerin; High-BMI, chemerin/adiponectin HMW; and Low-BMI, IL-8.

**Table 1:** Effect of each feature selected of model #5 for each of the group (Total cohort, High-BMI, and Low-BMI) with 2 to 5 features.

Model	Total cohort	BMI	Gender	Age	Adipsin/Chemerin	Adipsin/CRP
	High-BMI	BMI	Gender	Age	Adipsin/CRP	Chemerin/Adinopectin_HMW
	Low-BMI	BMI	Gender	Age	IL-8	Adipsin/CRP
5 variables	M1	■	■	■	■	■
4 variables	M2	■	■	■	■	
	M3	■	■	■		■
	M4	■	■		■	■
	M5	■		■	■	■
	M6	■	■	■	■	■
3 variables	M7	■	■	■		
	M8	■	■		■	
	M9	■	■			■
	M10	■		■	■	
	M11	■		■		■
	M12	■			■	■
	M13		■	■	■	
	M14		■	■		■
	M15			■	■	■
2 variables	M16	■	■			
	M17	■		■		
	M18	■			■	
	M19	■				■
	M20		■	■		
	M21		■		■	
	M22		■			■
	M23			■	■	
	M24			■		■
	M25				■	■

Adiponectin_HMW, Adiponectin high molecular weight; BMI, bone mass index; CRP, C-reactive protein; IL-8, interleukin 8. M refers to the model. Symbol ■ shows the selected parameter in the model.

**Figure 3:**
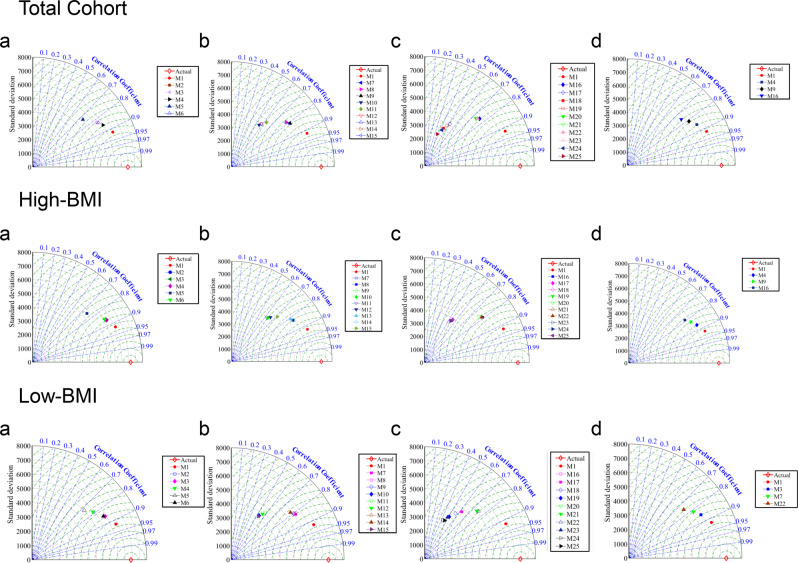
Taylor diagram to find the best sub-variables of models in testing stage with 2 to 5 variables for the Total cohort; High-bone mass index (BMI) and Low-BMI for M1-M25 as defined in Table 1. Model (M) with (**a**) 4 variables; (**b**) 3 variables; (**c**) 2 variables, and (**d**) best of all the models. Actual refers to the calculated infrapatellar fat pad volume.

Data showed for the Total cohort (Fig. 3 Total Cohort a) within the models with 4 variables (M2-M6), that the lowest performance was related to the model that comprises BMI, age, adipsin/chemerin and adipsin/CRP. This means that gender was the most important among the 5 selected variables. After M1, the best result was obtained for M4, which shows that age has the lowest effect. Among those with 3 sub-variables (M7-M15) (Fig. 3 Total Cohort b), the best included the variables BMI, gender and adipsin/CRP, while not using gender and adipsin/CRP (two of three variables in the best selected ones) led to the worst model. Among the models with 2 sub-variables (M16-M25) (Fig. 3 Total Cohort c), the best included BMI and gender and the worst adipsin/CRP and adipsin/chemerin. A comparison of M1 with those consisting of 2 sub-variables (Fig. 3 Total Cohort d) indicated that fewer variables result in weaker model performance. Thus, the most effective variables were gender and BMI for this group.

For the High-BMI, the results at Fig. 3 indicated that gender is the most effective variable in IPFP volume prediction. Hence, not using this variable leads to the worst model (Fig. 3 High-BMI a). The lowest effect is from age, as the model including BMI, gender, adipsin/CRP, chemerin/adiponectin HMW performed the best with four variables (Fig. 3 High-BMI a). From the models with 3 variables (Fig. 3 High-BMI b), removing gender and adipsin/CRP result in the highest error in IPFP volume prediction. Adipsin/CRP was selected as the most effective variable in the models with 3 variables (Fig. 3 High-BMI b) and using only BMI and adipsin/CRP results in the worst model (Fig. 3 High-BMI b). As shown in Fig. 3 (High-BMI c), not using risk factors as variables leads to the worst model. Therefore, it can be concluded that although adipsin/CRP is important, gender demonstrated a higher significance. Similar to the data of the Total cohort, the High-BMI showed that less variables lead to higher error in IPFP volume prediction (Fig. 3 High-BMI d).

Likewise, for the Low-BMI group (Fig. 3 Low-BMI), gender was selected as the most effective variable in IPFP volume prediction. Accordingly, the combination of BMI and gender comprises the most effective sub-variables of models with 2 variables (Fig. 3 Low-BMI c), and not using these variables simultaneously resulted in the worst model with 3 sub-variables (Fig. 3 Low-BMI b). In models with 2 variables (Fig. 3 Low-BMI c), gender and adipsin/CRP were selected as the best sub-variables. As for the other groups, less variables resulted in lower performance.

Altogether, these data revealed that for each of the three groups studied, gender was the most effective, followed by adipsin/CRP.

### Impact of gender separation on IPFP volume prediction

**Figure 4:**
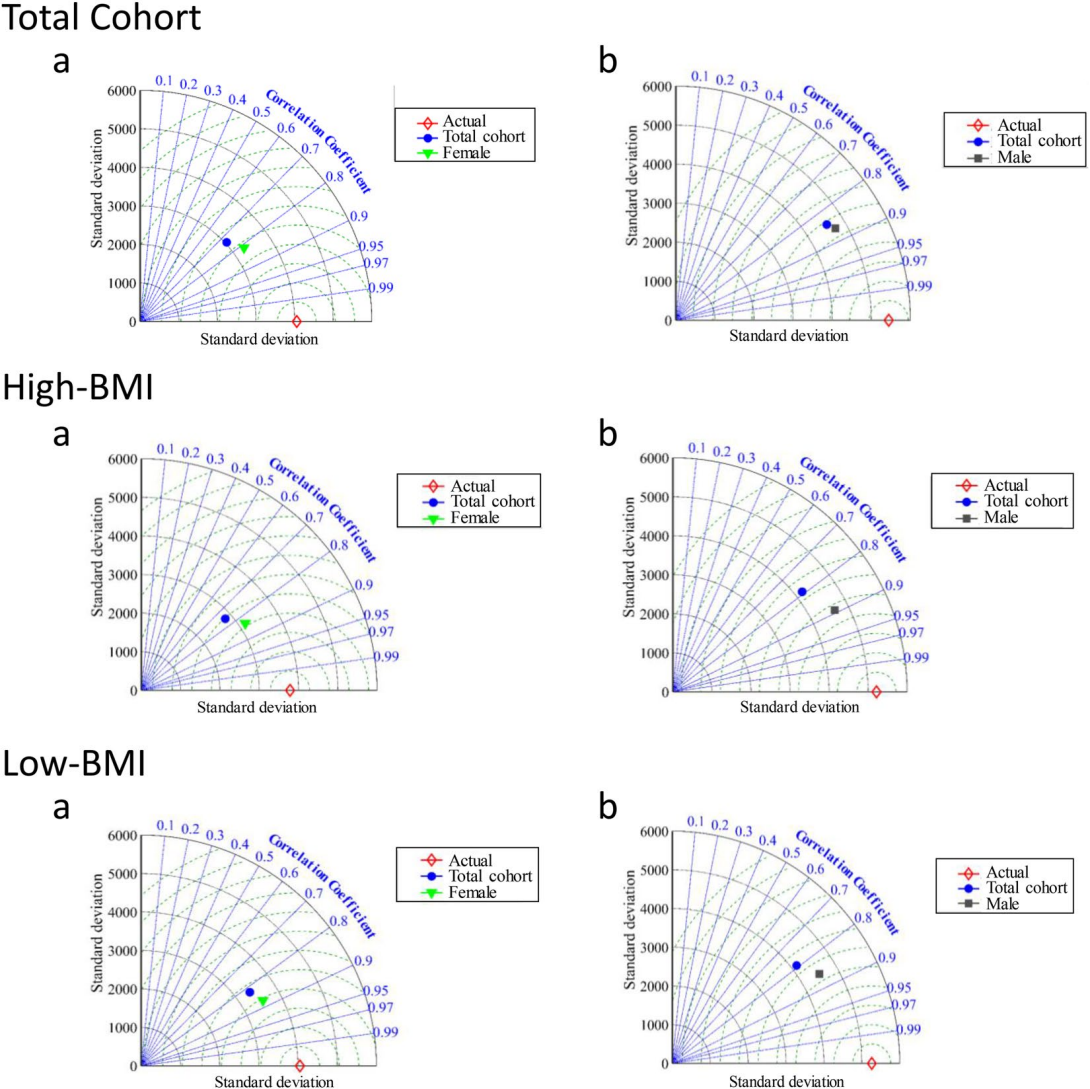
Taylor diagram for Total Cohort, High-bone mass index (BMI) and Low-BMI in testing stage analyzing the performance of their respective model #5 (as defined in the Supplementary Table S4) based on gender separation. (**a**) are diagrams of the female and (**b**) male.

**Figure 5:**
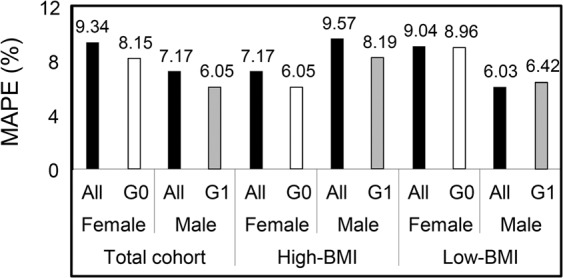
Comparison of the mean absolute percentage error (MAPE) effect of the gender separation on the Total cohort, High-bone mass index (BMI), and Low-BMI in testing stage. All, indicates studied gender from whole dataset and G0, female and G1, male.

As previous literature indicates that IPFP volume is significantly greater in male compared to female individuals^35–37^, and the previous section demonstrates that gender is the most important variable, we thus examined the impact of gender. Each group was further separated by female and male and their respective model #5 (Supplementary Table S4) was tested for IPFP volume prediction using the ANFIS-FCM. As illustrated in Fig. 4, the effect of gender separation for Total cohort, High-BMI and Low-BMI represented in Taylor diagrams confirm for all groups the superior performance of the model that considers gender separation compared to the main one comprising female and male together. Figure 5 illustrates the MAPE results of the modeling, taking gender. Data showed that gender separation yields superior performance compared to the original model for the Total cohort and High-BMI. In the Low-BMI, both gender and BMI-based was about similar for the females, but for the males, the BMI-based model made for a slightly better performance in IPFP volume prediction. The MAPE values were improved 13% and 16% for the Total cohort, 16% and 14% for the High-BMI for female and male respectively.

As illustrated in Supplementary Table S5, the WUB demonstrated that the Total cohort had the best statistical indices with gender separation (i.e. lower of each of these indices), indicating that gender separation leads to a model superior to the original for reliable forecasting of IPFP volume value.

ANFIS-FCM based pseudocodes (algorithms) for IPFP volume prediction based on gender separation were then generated for the Total cohort (Supplementary Materials).

### Reproducibility of the proposed model

In order to evaluate the reproducibility of the developed ANFIS-FCM gender-based models, a dataset of OA patients from a clinical trial for Licofelone/Naproxen, was employed^31^. Patients from this cohort, compared to the OAI participants, had a higher percentage of females and higher WOMAC subscores (Supplementary Table S1a).

According to the above data in which the Total cohort with gender separation had better WUB values than those with using BMI separation, the Licofelone/Naproxen cohort was separated according to gender.

**Figure 6:**
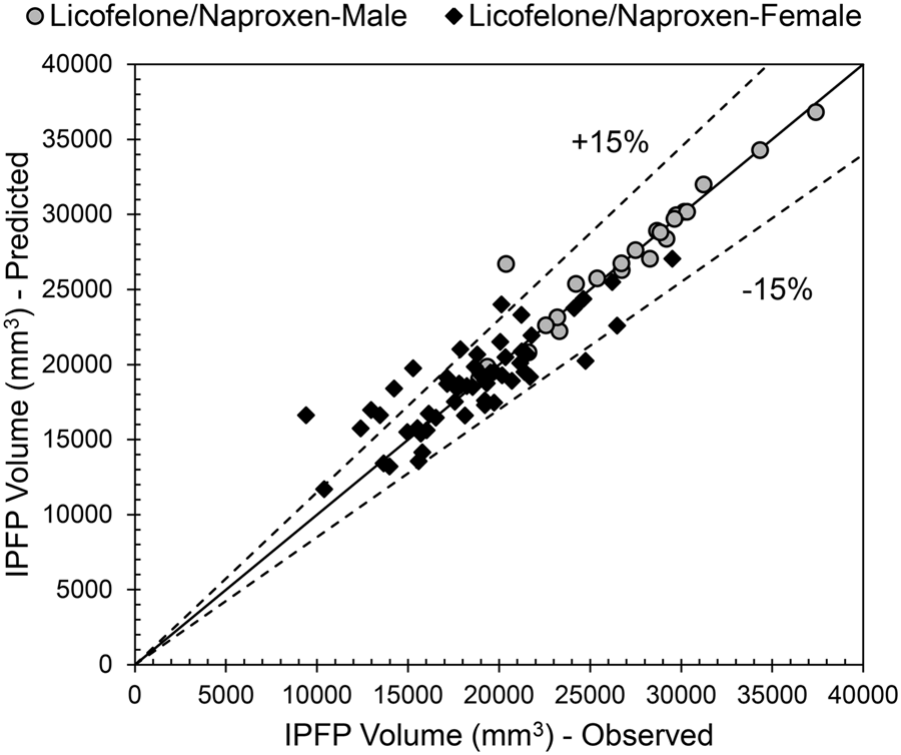
Scatter plot of infrapatellar fat pad (IPFP) volume prediction by the developed neuro-fuzzy inference system embedded with fuzzy c-means clustering pseudocodes for the Licofelone/Naproxen cohort according to gender separation.

**Figure 7:**
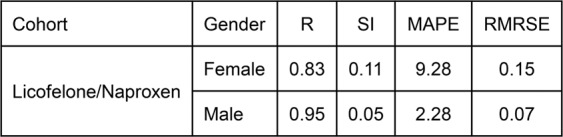
Statistical indices of infrapatellar fat pad (IPFP) volume prediction by the developed neuro-fuzzy inference system embedded with fuzzy c-means clustering pseudocodes for the Licofelone/Naproxen cohort according to gender separation. R, correlation coefficient; SI, scatter index; MAPE, mean absolute percentage error; RSMRE, root mean square relative error.

Fig. 6 shows the scatter plot and statistical indices for the predicted IPFP volume vs. observed data for the Naproxen/Licofelone female and male cohorts using the described pseudocodes (Supplementary Materials). Data revealed that most data are located around the exact line and in the ±15% error range (Fig. 6), in addition to both genders having a high R (female, 0.83; male, 0.95) and low RMSRE (0.15 and 0.07, respectively) (Fig. 7). Therefore, the proposed ANFIS-FCM pseudocodes for the IPFP volume prediction are highly reliable and generalizable.

## Discussion

Early detection of OA progressors is crucial but remains a challenge. This study is the first step aiming toward this goal. Hence, as IPFP is now considered an early marker of OA incidence or progression^5–9^, being able to predict this tissue volume with serum biomarkers could enable a next step in which this information will be used to predict/discriminate OA progressors from non-progressors.

Here, a comprehensive approach employing a machine learning method was carried out with some adipokines/related inflammatory factors and their ratios to predict the IPFP volume of OA patients. Such a technique was chosen due to the high amount of sub-variables, as it would have been difficult to investigate all the possible input combinations with traditional statistics modeling. Findings demonstrate that the combination of the serum levels of some adipokines/inflammatory factors and the three main OA risk factors predicted with high accuracy and reproducibility the IPFP volume. More specifically, for each group studied, the best model included only 5 variables for predicting the IPFP volume in which, in addition to age, gender, BMI and adipsin/CRP, the factor/ratio for each group was for Total cohort, adipsin/chemerin; High-BMI, chemerin/adiponectin HMW; and L-BMI, IL-8. The fact that age, gender and BMI were included in the best models for each group is not surprising, as in addition to being the three main risk factors for OA, each of them was also found to be associated with IPFP size^35,38,39^. However, further investigation of the impact of each variable on IPFP volume prediction in each group indicated that gender was the most effective variable and data separation based on gender improved the prediction results compared to the BMI-based models. For each gender, we then generated an ANFIS-FCM pseudocode with the 5 variables for predicting IPFP volume, in the form of an evolutionary computation equation. Notably, and of high clinical significance, the reproducibility experiment performed with symptomatic OA patients from a clinical trial and representing OA patients with a higher disease severity (higher WOMAC), then a real life scenario, demonstrated excellent coefficient correlation and statistical indices for each gender.

The finding that each model comprises at least a ratio of the serum levels of adipokines/inflammatory factors corroborates the usefulness of ratios as valuable and reliable predictive tools. Although the selected models should be used as a combination of the 5 indicated variables, it is interesting to note that, for each group studied; the adipsin/CRP ratio was included and is the most important one after gender. This indicates that this particular ratio is a suitable marker for the disease and not only for a particular sub-group.

Moreover, it is suggested that a ratio is a better predictor when both factors have different roles in a given disease process. This is in line with the ratios chosen as the best model for each group. Hence, in OA, adipsin and CRP were reported to present distinct roles. Adipsin is a structural component of the alternative complement pathway, involved in energy metabolism, associated in OA with cartilage volume loss and suggested to be involved in this tissue’s catabolic process^12,40–42^. In contrast, CRP is a systemic marker of inflammation. The other factors selected as a ratio for the Total cohort, adipsin/chemerin, also have a different role, as chemerin appears to be an inflammatory factor^43,44^ in addition to being found associated with the disease activity of an inflammatory (auto-immune) arthritis disease: rheumatoid arthritis^45^. Likewise, for chemerin/adiponectin HMW selected in the High-BMI group, although chemerin is considered an inflammatory factor, adiponectin demonstrated a protective role in OA articular tissues^46^. In addition, these two adipokines were found to contribute reciprocally to the development of metabolic syndrome^47^, a syndrome which suggested to contribute to some extent to the development of OA^48,49^.

IL-8 was the only individual factor included in a model and was found for the Low-BMI group. This interleukin is considered a pro-inflammatory mediator and acts as a chemoattractant for neutrophils^50,51^, with an increased expression in OA^52,53^. Interestingly, IL-8 has recently been associated with signal intensity alteration in the IPFP of OA patients^54^, although its specific role in such alteration remains to be identified.

Data also revealed that the main form of the ratio used for the model was justified, as the use of the inverse form slightly decreased the performance of the model in both training and testing stages.

In addition to the models and the pseudocodes generated for each gender, this study has two other major strengths. First, as no direct artificial intelligence technologies exist for the amount of the sub-variables, 2.81E + 14, to be tested to identify the best model for each group, we developed a selection approach based on PSO to find the best sub-variables for IPFP volume. Second, for the validation of the models, a cohort of symptomatic knee OA patients from a Canadian clinical trial was used, establishing the generalizability of our prediction models, at least in North America, as the participants used to generate the pseudocodes were from the United States.

By using the models for each gender and the pseudocodes for OA patients provided in this study, the next step will be to develop a predictive IPFP volume tool for OA progressors. Such a prediction model will be of importance not only in identifying disease status, but also for optimal patient management, as well as for the stratification of OA patients for drug discovery.

As in all studies, there are limitations. First, we chose a given panel of adipokines and related inflammatory factors that have been associated with OA. However, many other adipokines/related inflammatory factors have been reported and could be further tested. One could question the use of adipokines as biomarkers. Although there are several reports and many biomarkers being tested for OA progression, to date, none has been found sufficiently discriminating for an accurate diagnostic or predict prognosis; therefore, we elected to choose factors other those that are usually tested. However, adipokines were not chosen randomly, but selected, since their major source is the IPFP. Thus, upon production, these factors could be released early into the joint as well as in the circulation. Another limitation of this study is that we could have selected other articular structures such as the cartilage degradation as the outcome instead of the IPFP size. The reason for not having chosen the cartilage is that such an association, with the factors under study, was already done by our group^12^, but more importantly, this tissue alteration does not occur early during the disease process. Other articular structures could have been selected; however, as with the cartilage, for the majority of them their alterations were not occurring at early stages. In contrast, the IPFP has been reported to be associated not only with the progression of OA, but also with its onset; thus, before measurable cartilage degeneration and many of other articular structure alteration. Finally, among all other IPFP parameters that could have been used as an outcome instead of the volume, were the tissue area and signal intensity (hypointense/hyperintense)^5,7,37,55–57^. However, to date the methods used for the evaluation of those parameters have not yet been fully standardized nor a consensus established. In regard to the IPFP volume, as determine in the present work, this measure is quantitative and from the perspective of creating a diagnosis/prognostic tool, the volume can be assessed from the images of the T2 weighted fat-saturated sequence commonly included in a standard diagnostic MRI exam of the knee.

In conclusion, we have developed an evolutionary-based variable method for selecting the best variables combinations in IPFP volume prediction, using a panel of adipokines/related inflammatory factors, state-of-the-art machine learning methodologies in which the IPFP size determination can be obtained with high accuracy and based on only 5 variables, which was confirmed with an external cohort from a clinical trial. Moreover, this study also provides pseudocodes of IPFP volume prediction for each gender.

## Supplementary information

Supplementary information.

Supplementary information 2.

Supplementary information 3.

## Data Availability

Data used for this study were from the OAI database, which is publicly available online (https://nda.nih.gov/oai/). The data sets generated and/or analyzed during the current study are included in this published article or available from the corresponding author on reasonable request.
