# Supplementary material for: Serum adipokines/related inflammatory factors and ratios as predictors of infrapatellar fat pad volume in osteoarthritis: Applying comprehensive machine learning approaches
Source: Sci Rep. 2020 Jun 19;10:9993. doi: 10.1038/s41598-020-66330-0 (PMC7305166; doi:10.1038/s41598-020-66330-0)
Supplement: Supplementary file 1 — Supplementary information. [file 41598_2020_66330_MOESM1_ESM.pdf]

Serum adipokines/related inflammatory factors and ratios as predictors of infrapatellar fat pad volume in osteoarthritis: Applying comprehensive machine learning approaches

Hossein Bonakdari, PhD, Ginette Tardif, PhD, François Abram, PhD, Jean-Pierre Pelletier, MD, Johanne Martel-Pelletier, PhD

Supplementary Tables

Table S1a. Participant characteristics

	Total cohort* (n=678)	Reproducibility (n=80)	p-value
Age, years	61 ± 9	62 ± 8	0.332
Gender, male, % (n)	57% (388)	29% (23)	<0.0001**
BMI, kg/m^2	30.3 ± 4.7	29.0 ± 4.1	0.050
WOMAC			
Pain (0-20)	3.8 ± 4.0	11.4 ±2.5	<0.0001
Function (0-68)	12.9 ± 12.7[a]	36.1 ±12.7	<0.0001
Stiffness (0-8)	2.1 ± 1.8[b]	4.9 ± 1.7	<0.0001
Total (0-96)	18.8 ± 17.8[c]	52.5±15.5	<0.0001
Kellgren-Lawrence grade[d], % (n)			
0-1	28% (192)		
2	33% (225)		
3	29% (198)		
4	9% (62)		
Joint space width, mm	3.96 ± 1.64[e]		
Infrapatellar Fat Pad volume, mm^3	26,938 ± 6,858	20,935 ± 5,558	<0.0001
Biomarker levels (median [IR])			
Adiponectin HMW, μg/ml	3.5 (2.2, 5.6)	4.5 (3.3, 6.7)	<0.0001
Adiponectin LMW, μg/ml	2.1 (1.6, 2.8)		
Adipsin, μg/ml	7.2 (5.8, 8.9)	4.1 (3.5, 4.6)	<0.0001
Chemerin, ng/ml	6.4 (5.1, 7.8)	8.1 (6.6, 9.9)	<0.0001
CRP, μg/ml	2.0 (0.9, 4.1)	2.8 (1.1, 6.4)	0.033
IL-8, pg/ml	11.3 (8.7, 14.5)	16.1 (11.4, 19.7)	<0.0001
Leptin, ng/ml	15.3 (7.5, 32.8)		
MCP-1, pg/ml	422.1 (311.0, 544.0)		
Visfatin, pg/ml	292.0 (4.7, 737.7)		

Table S1b. Infrapatellar Fat Pad Volume Assessment

	Method Development* (n=112)	Validation* (n=38)	p-value
Age, years	59 ± 9	61 ± 10	0.573
Gender, male, % (n)	49% (55)	55 (21)	0.512**
BMI, kg/m^2	27.5 ± 5.0	30.0 ± 5.5	**0.011**
Subcohort			
Control	36% (40)	26% (10)	
Progression	64% (72)	74% 28	0.288**
WOMAC (median [IR])			
Pain (0-20)	1.0 (0.0, 4.5)	1.0 (0.0, 5.0)	0.852
Function (0-68)	4.0 (0.0, 17.0)[a]	4.2 (0.0, 16.0)	0.968
Stiffness (0-8)	1.0 (0.0, 3.0)	2.0 (0.0, 3.0)	0.720
Total (0-96)	8.0 (0.0, 23.0)[a]	8.7 (0.0, 24.0)	0.807
Kellgren-Lawrence grade, % (n)			
0-1	52% (58)[a]	40 (15)	
2	26% (29)[a]	18% (7)	
3	18% (20)[a]	34% (13)	
4	4% (4)[a]	8% (3)	0.098**
Joint space width, mm	4.24 ± 1.36[b]	4.25 ± 1.59	0.643
Infrapatellar Fat Pad volume, mm^3	26,421 ± 6,263	26,628 ± 6,075	0.642
Biomarker levels (median [IR])			
Adiponectin HMW, μg/ml	3.9 (2.8, 6.7)	4.4 (2.7, 5.7)	0.971
Adiponectin LMW, μg/ml	2.2 (1.7, 2.9)	2.1 (1.6, 2.9)	0.957
Adipsin, μg/ml	6.5 (5.6, 8.4)	6.9 (5.8, 8.5)	0.621
Chemerin, ng/ml	5.9 (4.2, 7.2)	5.7 (4.7, 6.8)	0.875
CRP, μg/ml	1.2 (0.5, 3.4)	1.6 (0.9, 3.3)	0.176
IL-8, pg/ml	11.0 (8.6, 14.2)[a]	10.8 (8.2, 13.4)	0.621
Leptin, ng/ml	11.7 (4.8, 23.2)	11.8 (7.1, 39.4)	0.244
MCP-1, pg/ml	424.7 (307.1, 549.4)	401.1 (276.2, 500.8)	0.188
Visfatin, pg/ml	58.6 (0.0, 595.3)	153.5 (0.0, 458.0)	0.753

*Individuals were from the Osteoarthritis Initiative (OAI) cohort (http:\\oai.nih.gov), and for the Reproducibility, they were from the Licofelone/Naproxen clinical trial cohort (1).

Data are presented as mean ± SD, % and number (n) of patients, or median and interquartile [IR].

Continuous variables were compared using the Student's t-test/Mann-Whitney test; **proportions were compared using the chi-squared test/Fisher's exact test; p-values ≤ 0.050 were considered statistically significant.

Missing values: a) 9; b) 2; c) 11; d) 1; e) 3

Adiponectin HMW, Adiponectin high molecular weight; Adiponectin LMW, Adiponectin low molecular weight; BMI, body mass index; CRP, C-reactive protein; IL-8, interleukin 8; IR, interquartile range; MCP-1, monocyte chemoattractant protein-1; WOMAC, Western Ontario and McMaster Universities Osteoarthritis Index.

Table S2. Possible sub-features of models with 1 to 48 features

									Sum
No. of inputs	1	2	3	4	5	6	7	8	-
No. of sub-features	4.80E+01	1.13E+03	1.73E+04	1.95E+05	1.71E+06	1.23E+07	7.36E+07	3.77E+08	4.65E+08
No. of inputs	9	10	11	12	13	14	15	16	-
No. of sub-features	1.68E+09	6.54E+09	2.26E+10	6.97E+10	1.93E+11	4.82E+11	1.09E+12	2.25E+12	4.12E+12
No. of inputs	17	18	19	20	21	22	23	24	-
No. of sub-features	4.24E+12	7.31E+12	1.15E+13	1.67E+13	2.23E+13	2.74E+13	3.10E+13	3.22E+13	1.53E+14
No. of inputs	25	26	27	28	29	30	31	32	-
No. of sub-features	3.10E+13	2.74E+13	2.23E+13	1.67E+13	1.15E+13	7.31E+12	4.24E+12	2.25E+12	1.23E+14
No. of inputs	33	34	35	36	37	38	39	40	-
No. of sub-features	1.09E+12	4.82E+11	1.93E+11	6.97E+10	2.26E+10	6.54E+09	1.68E+09	3.77E+08	1.87E+12
No. of inputs	41	42	43	44	45	46	47	48	-
No. of sub-features	7.36E+07	1.23E+07	1.71E+06	1.95E+05	1.73E+04	1.13E+03	4.80E+01	1.00E+00	8.78E+07
All possible input sub-features									**2.81E+14**

The sub-features were determined using Particle Swarm Optimization (PSO) algorithm combined with an artificial neural network and Monte-Carlo simulation.

Table S3. The correlation coefficient of the variables and the infrapatellar fat (IPFP) volume

	BMI	Gender	Age	MCP-1	IL-8	Visfatin	Leptin	Chemerin	CRP	Adipsin	Adiponectin HMW	Adiponectin LMW	IPFP volume
BMI	1	-0.06	-0.14	-0.07	-0.08	0.00	0.50	0.21	0.25	0.29	-0.23	-0.22	0.15
Gender	-	1	0.01	-0.03	-0.04	0.01	-0.47	-0.18	-0.17	-0.04	-0.25	-0.14	0.58
Age	-	-	1	0.09	0.13	0.08	-0.02	0.04	-0.01	0.26	0.17	0.12	-0.07
MCP-1	-	-	-	1	0.22	0.02	-0.01	0.07	-0.03	0.03	0.16	0.16	-0.05
IL-8	-	-	-	-	1	-0.02	-0.03	0.03	0.07	-0.02	0.04	0.01	-0.02
Visfatin	-	-	-	-	-	1	-0.03	0.00	0.05	0.05	-0.03	0.00	-0.02
Leptin	-	-	-	-	-	-	1	0.20	0.22	0.30	0.02	0.02	-0.21
Chemerin	-	-	-	-	-	-	-	1	0.13	0.24	0.08	0.04	0.01
CRP	-	-	-	-	-	-	-	-	1	0.13	-0.11	-0.09	-0.03
Adipsin	-	-	-	-	-	-	-	-	-	1	0.11	0.10	0.01
Adiponectin HMW	-	-	-	-	-	-	-	-	-	-	1	0.58	-0.17
Adiponectin LMW	-	-	-	-	-	-	-	-	-	-	-	1	-0.15
IPFP volume	-	-	-	-	-	-	-	-	-	-	-	-	1

Adiponectin HMW, Adiponectin high molecular weight; Adiponectin LMW, Adiponectin low molecular weight; BMI, body mass index; CRP, C-reactive protein; IL-8, interleukin 8; MCP-1, monocyte chemoattractant protein-1

Table S4. Results of the developed algorithm particle swarm optimization (PSO)-based feature selection (PSOBVS) Total Cohort

FS	1	2	3	4	5	6	7	8	9	10	11	12	13	14	15	16	17	18	19	20	21	22	23	24	25	26	27	28	29	30	31	32	33	34	35	36	37	38	39	40	41	42	43	44	45	46	47	48
1																																																
2																																																
3																																																
4																																																
5																																																
6																																																
7																																																
8																																																
9																																																
10																																																
11																																																
12																																																
13																																																
14																																																
15																																																
16																																																
17																																																
18																																																
19																																																
20																																																
21																																																
22																																																
23																																																
24																																																
25																																																
26																																																
27																																																
28																																																
29																																																
30																																																
31																																																
32																																																
33																																																
34																																																
35																																																
36																																																
37																																																
38																																																
39																																																
40																																																
41																																																
42																																																
43																																																
44																																																
45																																																
46																																																
47																																																
48																																																

Table S4. High-BMI

F S	1	2	3	4	5	6	7	8	9	10	11	12	13	14	15	16	17	18	19	20	21	22	23	24	25	26	27	28	29	30	31	32	33	34	35	36	37	38	39	40	41	42	43	44	45	46	47	48
1																																																
2																																																
3																																																
4																																																
5																																																
6																																																
7																																																
8																																																
9																																																
10																																																
11																																																
12																																																
13																																																
14																																																
15																																																
16																																																
17																																																
18																																																
19																																																
20																																																
21																																																
22																																																
23																																																
24																																																
25																																																
26																																																
27																																																
28																																																
29																																																
30																																																
31																																																
32																																																
33																																																
34																																																
35																																																
36																																																
37																																																
38																																																
39																																																
40																																																
41																																																
42																																																
43																																																
44																																																
45																																																
46																																																
47																																																
48																																																

Table S4. Low-BMI

FS	1	2	3	4	5	6	7	8	9	10	11	12	13	14	15	16	17	18	19	20	21	22	23	24	25	26	27	28	29	30	31	32	33	34	35	36	37	38	39	40	41	42	43	44	45	46	47	48
1																																																
2																																																
3																																																
4																																																
5																																																
6																																																
7																																																
8																																																
9																																																
10																																																
11																																																
12																																																
13																																																
14																																																
15																																																
16																																																
17																																																
18																																																
19																																																
20																																																
21																																																
22																																																
23																																																
24																																																
25																																																
26																																																
27																																																
28																																																
29																																																
30																																																
31																																																
32																																																
33																																																
34																																																
35																																																
36																																																
37																																																
38																																																
39																																																
40																																																
41																																																
42																																																
43																																																
44																																																
45																																																
46																																																
47																																																
48																																																

FS indicates feature selection. Numbers in the rows and columns indicates (1-3), risk factors variables; (4-12), biomarkers values; (13-48), biomarkers ratios values.

1=BMI, body mass index (kg/m^2); 2=Gender; 3=Age; 4=MCP-1, monocyte chemoattractant protein-1 (pg/ml); 5=IL-8, interleukin 8 (pg/ml); 6=Visfatin (pg/ml); 7=Leptin (ng/ml); 8=Chemerin (ng/ml); 9=CRP, C-reactive protein (μg/ml); 10=Adipsin (μg/ml); 11= Adiponectin HMW, Adiponectin high molecular weight (μg/ml); 12= Adiponectin LMW, Adiponectin low molecular weight (μg/ml); 13= Adiponectin HMW/Adiponectin LMW; 14= Adiponectin HMW/MCP-1 [10^{-3}]; 15= Adiponectin LMW/MCP-1 [10^{-3}]; 16= Adipsin/Adiponectin HMW; 17= Adipsin/Adiponectin LMW; 18= Adipsin/Chemerin; 19= Adipsin/CRP; 20= Adipsin/IL-8; 21= Adipsin/Leptin; 22= Adipsin/MCP-1 [10^{-3}]; 23= Adipsin/Visfatin [10^{-3}]; 24= Chemerin/Adiponectin HMW; 25= Chemerin/Adiponectin LMW; 26= Chemerin/Il-8; 27= Chemerin/MCP-1 [10^{-3}]; 28= Chemerin/Visfatin [10^{-2}];29= CRP/Adiponectin HMW; 30= CRP/Adiponectin LMW; 31= CRP/Cemerin; 32= CRP/IL-8; 33= CRP/MCP-1 [10^{-3}]; 34= CRP/Visfatin [10^{-3}]; 35= IL-8/Adiponectin HMW ; 36= IL-8/Adiponectin LMW; 37= Il-8/MCP-1 [10^{-3}]; 38= IL-8/Visfatin [10^{-2}]; 39= Leptin/Adiponectin HMW; 40= Leptin/Adiponectin LMW; 41= Leptin/Chemerin; 42= Leptin/CRP; 43= Leptin/IL-8; 44= Leptin/MCP-1 [10^{-3}]; 45= Leptin/Visfatin; 46= Visfatin/Adiponectin HMW; 47= Visfatin/Adiponectin LMW ; 48=Visfatin/MCP-1 [10^{-2}]. Of note, the ratio were calculated employing natural log (ln) transformation to reduce the effect of skew.

Table S5. Uncertainty data for the gender separation models

BMI	Gender	Standard deviation of Prediction Error	95% Prediction Error Interval		Width of Uncertainty Band (WUB)
Total cohort	All (G0)	2578.79	(-269.56	269.56)	±269.56
	G0	2501.81	(-311.57	211.45)	±261.51
	All (G1)	3089.73	(-314.85	331.08)	±322.97
	G1	2917.33	(-372.87	237.02)	±304.95
High-BMI	All (G0)	2271.39	(-354.22	306.54)	±330.38
	G0	2111.55	(-307.94	306.32)	±307.13
	All (G1)	3098.35	(-418.71	430.44)	±424.57
	G1	3171.47	(-430.69	438.49)	±434.59
Low-BMI	All (G0)	2426.35	(-216.74	517.99)	±367.36
	G0	2591.85	(-390.66	394.19)	±392.42
	All (G1)	2953.84	(-249.11	550.72)	±399.91
	G1	2832.53	(-359.05	407.93)	±383.49

All, whole cohort for female (G0) and male (G1); BMI, bone mass index.

REFERENCES

1. Raynauld JP, Martel-Pelletier J, Bias P, Laufer S, Haraoui B, Choquette D, et al. Protective effects of licofelone, a 5-lipoxygenase and cyclo-oxygenase inhibitor, versus naproxen on cartilage loss in knee osteoarthritis: a first multicentre clinical trial using quantitative MRI. Ann Rheum Dis. 2009;68(6):938-47.
