# Supplementary material for: Serum adipokines/related inflammatory factors and ratios as predictors of infrapatellar fat pad volume in osteoarthritis: Applying comprehensive machine learning approaches
Source: Sci Rep. 2020 Jun 19;10:9993. doi: 10.1038/s41598-020-66330-0 (PMC7305166; doi:10.1038/s41598-020-66330-0)
Supplement: Supplementary file 2 — Supplementary information 2. [file 41598_2020_66330_MOESM2_ESM.pdf]

Serum adipokines/related inflammatory factors and ratios as predictors of infrapatellar fat pad volume in osteoarthritis: Applying comprehensive machine learning approaches

Hossein Bonakdari, PhD, Ginette Tardif, PhD, François Abram, PhD, Jean-Pierre Pelletier, MD, Johanne Martel-Pelletier, PhD

Supplementary Materials

METHODS

Target Knee

Participants had symptomatic radiographic osteoarthritis (OA) and had undergone magnetic resonance imaging (MRI) at baseline for the target knee, and are defined as follows: i) if only one knee had evidence of radiographic OA; ii) if both knees had evidence of OA, the most symptomatic knee, based on the highest Western Ontario and McMaster Universities Osteoarthritis Index (WOMAC) pain score at baseline; iii) if the participant did not have evidence of radiographic OA, then the dominant knee, defined by the leg used to kick a ball, was used as the target knee.

Biomarker determination

All biomarkers were determined with specific assays according to the manufacturers' specifications as previously described[1]. The high (HMW) and low (LMW) molecular weight forms of adiponectin were determined by ELISA (Alpco, Salem, NH, USA) according to the manufacturer's specifications (dilution 1/5151**)**, and the data analyzed using the Thermo Multiscan Spectrum apparatus and the SkanIt software (Thermo Fisher Scientific, Waltham, MA, USA). Adipsin, C-reactive protein (CRP) (both at 1/3000 dilution, Milliplex MAP kit, EMD Millipore Corporation, Billerica, MA, USA), leptin, chemerin, interleukin (IL)-8, monocyte chemoattractant protein-1 (MCP-1) (each at 1/2 dilution, Luminex assay, R&D systems, Minneapolis, MN, USA) and visfatin (undiluted, Bio-Plex Pro Assay, Bio-Rad Laboratories,

Mississauga, ON, Canada) were quantitated by Luminex technology using the LiquiChip 200 apparatus (Qiagen, Toronto, ON, Canada) and the data analysis performed with the LiquiChip Analyzer software (Qiagen). For each biomarker, an 8-point standard curve and appropriate controls were included, and samples were done in duplicate.

The minimum detectable doses were for adiponectin HMW and LMW, 19 pg/ml; adipsin, 44 pg/ml; chemerin, 69 pg/ml; CRP, 1 pg/ml; leptin, 10.2 pg/ml; IL-8, 1.8 pg/ml; MCP-1, 9.9 pg/ml; and visfatin, 37.5 pg/ml.

IPFP volume assessment using magnetic resonance imaging (MRI)

Image normalization: preprocessing

All the MR images were normalized in terms of intensity (I) and size before processing for training or classification to increase segmentation accuracy and to address computation issues (time and memory). The intensity histogram was employed as the basic information to compute lower and upper thresholds, which were then used to perform the normalization of the intensity levels. To this end, a five step processing was applied to compute a threshold T: i) the histogram was computed over a number of channels equal to the range between the minimum and the maximum image intensities plus 1 (max – min + 1); ii) a reset of the first channel of the histogram was done if it contained the highest peak of the histogram; iii) then, seven models M_n were fitted to the histogram, each being a weighted combination of Gaussian(a, b, c) distributions, from 2 to 8, $M_n = \sum_{i=1}^{n} a_i \times exp(-((x - b_i)/c_i)^2)$; iv). From these seven models, a single one was chosen with respect to two criteria: the models where either amplitude a or average b were a negative value were excluded. Among the remaining models, the one whose Gaussian highest amplitude was the nearest to the highest amplitude of the histogram was selected; v) finally, the computed threshold T is the maximum value of the following two values: first is the $mean + 3 \times std$ value of the

Gaussian with the highest average b value of the selected model; second is the $mean + 3 \times std$ of the Gaussian model that best fits the difference between the histogram and the selected model. The threshold T is used to normalize the range of image intensities from $[0, T]$ to $[0,255]$ using $I_{normalized} = 255 \times min(1, I_{original}/T)$. After the normalization of the intensity, the image was down-sampled to 256x256 pixels using a nearest neighbor algorithm.

Optimization of the workflow

The configuration used for the computation is as follows: BATCH_SIZE=3, TRAINING_EPOCHES=100, FOLD_COUNT=6, KERNEL=(2,2), PAD=(0,0) and ITERATION_NUMBER=100. First, the MR images of the reference population were manually labelled for the two classes, IPFP and KJB. Each labelled image was down-sampled to 256x256 pixels using a nearest neighbor algorithm. Each 2D TSE image was normalized as described above. The network was trained on 112 individuals with normalized MR images and corresponding labels, both 256x256 pixel images, as input. 3D probability maps were computed to introduce the influence of a neighborhood. Each 3D probability map results from the weighted average (0.2, 0.8 and 0.2) of the 2D probability map of the 2 neighbors and current image after the classification of all the images of a sequence. This process was done on the entire training population. The 3D probability maps were up-sampled by interpolation to the original resolution of the images (340x344). Then the computation of a probability threshold that optimizes the Dice coefficient[2] between automated and manual segmentation was performed. This probability threshold was computed as the average of the probabilities corresponding to the optimum Dice coefficient computed for all sequences separately.

Segmentation process

The final segmentation process for all new sequences included: i) the normalization of the images, as described above; ii) the classification, using the optimized CNN; and iii) the processing of the probability maps. After classification, the 3D probability map was computed from the 2D probability map for the IPFP as described above, up-sampled to the original size of the image using a bi-cubic interpolation algorithm, and the 3D probability was finally compared to the probability threshold to obtain the IPFP 3D object. The volume of the IPFP was then computed as the sum of each voxel times the elementary volume of a voxel as defined in the image DICOM header and expressed as mm^3.

Validation of the automated IPFP volume assessment using CNN

Validation was performed on 38 individuals. Participant characteristics (Supplementary Table S1b) were balanced between the individuals used for the methodology development and the validation; the only exception being that the BMI was slightly, but statistically significant, in the validation population.

The IPFP manual segmentation was performed as previously described[3] by a single reader who was trained by an expert musculoskeletal radiologist. The Pearson correlation between the IPFP volumes obtained with manual reading and with the MRI automated technology using the developed CNN methodology revealed an excellent coefficient correlation, R=0.90.

Particle Swarm Optimization (PSO) and PSO-based variable selection (PSOBVS)

PSO is a stochastic population-based optimization algorithm[4]. It is inspired by the social behavior of fish schools and bird flocks. To find food, such swarms follow a cooperative path, whereby each individual keeps altering the search pattern according to its learning experience and other swarm members. The main idea behind the PSO algorithm is derived from the artificial life theory and utilizing swarms to search a large space as the solution space of the objective function

(evolutionary process). To survey the behavior of artificial systems with life species in the construct of swarm-based algorithms such as PSO via computer, the following basic principles were considered[5]. 1) Diverse response: the swarm ought not constrain its way to urge the assets in a limit scope. 2) Adaptability: the swarm ought to alter its behavior when the alteration is commendable. 3) Stability: the swarm ought not alter its behavior with each environmental alteration. 4) Quality: the swarm ought to be able to perceive the quality alteration within the space and react to it. 5) Proximity: the swarm ought to be able to perform time and simple space computations. It should be noted that the second and third principles are the inverse sides of the same coin. The abovementioned principles comprise the main specifications of artificial life systems (ALS) and can therefore be used as a guide to presenting ALS.

A particle's velocity and position alterations due to environmental changes can be updated in the PSO algorithm. PSO meets the stipulations of quality and proximity. Moreover, the swarm's movement in PSO is not limited and the optimum solution is continuously explored within the potential solution space. Particles maintain stable movement in the PSO algorithm but alter their movement to reconcile the environmental space changes. Consequently, PSO-based particle swarms encounter the five abovementioned principles.

Each individual in the PSO, which is a swarm-based searching technique, is known as a particle. A particle is defined as a possible solution to the desired optimization problem because it can memorize the optimal velocity and position of the swarm. In each generation, the new position of every particle is calculated by adjusting the velocity of each dimension through combining the particles' information. Particles alter their conditions continuously in the d-dimensional search space until they attain an optimal or balanced condition, or go beyond the computing limits. The objective function is employed to connect all dimensions of the problem space.

Assuming N is the swarm size, the particle position and velocity are defined as $X_i=(x_{i1}, x_{i2}, \ldots, x_{iD})$ and $V_i=(v_{i1}, v_{i2}, \ldots, v_{iD})$ respectively, the optimal position from experience of each individual in the swarm (swarm's optimal position) is $P_g=(p_{g1}, p_{g2}, \cdot, p_{gD})$ and the optimal position from experience of each particle (individual's optimal position) is $P_i = (p_{i1}, p_{i2}, \cdot\cdot, p_{iD})$. P_i and P_g represent pbest and gbest respectively.

The optimal position of each individual is calculated as follows:

$$P_{i,t+1}^{d} = \begin{cases} x_{i,t+1}^{d} & if \quad f(X_{i,t+1}) < f(P_{i,t}) \\ P_{i,t}^{d} & otherwise \end{cases} \quad (1)$$

where *x* and *P* represent particle position and optimal position respectively. The velocity and position vectors are updated as follows:

$$v_{i,t+1}^{d} = w \times v_{i,t}^{d} + c_1 \times rand \times (p_{i,t}^{d} - x_{i,t}^{d}) + c_2 \times rand \times (p_{g,t}^{d} - x_{i,t}^{d}) \quad (2)$$

$$x_{i,t+1}^{d} = x_{i,t}^{d} + v_{i,t+1}^{d} \quad (3)$$

where *v* represents the particle velocity, *t* is the iteration number (*t*=1 to maximum iteration number -1), *i* denotes the particle number (*i*=1 to *N*), *rand* is a random value in [0, 1], c_1 and c_2 are two user-defined parameters known as personal and global learning coefficients respectively, P_i and P_g are *pbest* and *gbest* respectively, and *w* is the inertia weight. Equation 2 has three parts. The first part denotes the effect of the particle's previous velocity. The second part is known as "cognitive" because the particle's movement here is based on its own experience. The particle's movement in the third part is based on the particle's current experience and the experience of the other particles. This third part is known as the social part. Consequently, *c1* and *c2* are called cognitive and social learning factors, respectively. More details on PSO are given in previous studies[6-8].

Artificial neural network (ANN)

ANN is a computational approach that involves a few straightforward processing components called neurons[9]. ANN neurons are as a rule organized in three main layers: input, hidden and output. The input layer contains the independent variables to estimate the desired target variable that is provided in the output layer. Unlike the single input and output layers, there can be several hidden layers. It is noteworthy that an ANN model with only one hidden layer can estimate a nonlinear complex system. Increasing the number of hidden layers or the number of neurons in the hidden layer(s) may lead to overfitting. The purpose of the hidden layer is to determine a multi-dimensional development of the input layer, which can facilitate good mapping of the input and output layers. The input layer neurons are linked to the hidden layer neurons (HLNs), and these are connected to the output layer neuron(s). More details about ANNs are given by Ebtehaj and Bonakdari[10].

Due to existing uncertainty in ANN results, Monte-Carlo simulation (MSC) is utilized to overcome the uncertainty in ANN modeling. Therefore, the number of MCS runs must be defined before modeling begins. The number of runs considered was 1000 in all simulations. All ANN models were structured as a single-layer feedforward neural network. The training algorithm was Levenberg-Marquardt (LM) and the activation functions of the hidden and output layers considered were tansig and purelin, respectively. The maximum allowable number of hidden neurons (NHN) in ANN is calculated as follows[11]:

$$TrSa \geq NHN(NIP + 2) + 1 \rightarrow NHN \leq \frac{(TrSa - 1)}{NIP + 2} \quad (4)$$

where *NHN* denotes the number of hidden neurons, *TrSa* the training samples and *NIP* the number of input parameters (from 1 to 48).

The cost value is calculated using the Akaike Information Criterion (AIC) [12]:

$$AIC = n \times Ln(MSE) + 2 \times k \quad (5)$$

Here, *MSE* denotes the mean squared error, *N* represents the number of samples and *k* denotes the number of customizable parameters in each matrix in the ANN model.

After the cost function is defined, optimization begins in order to find the best sub-variables of models with 1 to 48 variables. For this purpose, not only must the PSO parameters be defined, but the number of desired variables and input-output data pairs should also be provided. The user-defined PSO parameters, including inertia weight, population size, iteration number and global and personal learning coefficients are 0.9, 100, 500, 2.05 and 2.05, respectively. The PSO-based optimization starts by initializing the velocity and position of each individual and calculating the global best (gbest) and personal best (pbest). Pbest and gbest are updated throughout the PSO optimization. The main PSO loop starts by updating the position and velocity of each individual using equations 2 and 3, respectively. In each iteration, pbest and gbest are updated after the position and velocity are updated.

Adaptive neuro-fuzzy inference system embedded with fuzzy c-means clustering (ANFIS-FCM)

With fuzzy logic, there is no systematic process for designing a fuzzy controller; hence, Jang[13] presented an ANFIS model that is able to combine the abilities of neural networks and fuzzy logic. For simplification, it is assumed that the ANFIS system has two inputs (*x* and *y*) and one output (*z*). The ANFIS architecture is a 5-layer system. The first layer is the input node and estimates the membership value belonging to each appropriate fuzzy set using the membership function. The maximum allowable number of clusters is calculated as follows[11]:

$$Max.MF \leq \frac{TrSa}{NIP \times 3 + 1} \quad (6)$$

where *Max.MF* denotes the maximum allowable number of membership functions, *TrSa* is the training samples and *NIP* is the number of input parameters that differs from 1 to 48.

In the second layer, the AND operator is used to represent the premise part of the fuzzy rules. The output of this layer is the product of the degrees of the first layer. The main objective of the third layer is to determine the ratio of the ith rule's weight to the total weight of all rules. As a result, w_i is obtained as the normalized weight. In the fourth layer, the weighted value of each fuzzy rule is calculated. The fifth layer, which is related to the output node, calculates the total output by collecting all input signals. Therefore, the defuzzification process in this layer deforms the results of each fuzzy rule to the defuzzification output. The final ANFIS network output is the average weighted output of all rules, which is calculated as follows:

$$Final \quad output = \frac{\sum_{i=1}^{N} w_i z_i}{\sum_{i=1}^{N} w_i} \tag{7}$$

where z represents the output, w is the weight, N is the number of rules in the fuzzy inference system (FIS).

The distinctive variable of ANFIS is the provision of a hybrid, learning algorithm as a combination of backpropagation (BP) and least square (LS). The BP method is used to adjust the nonlinear parameters in the premise part, while the LS method is used to determine the linear parameters of the consequent part.

ANFIS modeling begins with training through the BP and LS methods, prior to which, an initial FIS structure must be created. There are several methods of FIS generation, such as sub-clustering (SC), grid partitioning (GP) and FCM. Owing to the good performance of FCM, this method is used in the present study to generate FIS. More details about ANFIS are provided by Ebtehaj and Bonakdari[14].

FCM is presented based on the definition of different clusters. The main goal of clusters is to encourage the highest between-class interval and lowest in-class interval conceivable when classifying data. To avoid the validation issue with providing the number of clusters, FCM is exerted. The concept is as follows.

The central vector of the population samples ($\bar{x}$), membership matrix ($U^{(k)}$), clustering center matrix ($V^{(k+1)}$) and adaptive function of clustering-C (L(c)) are calculated with the following equations.

$$\bar{x} = \frac{1}{n}\sum_{i=1}^{c}\sum_{j=1}^{n} u_{ij}^{m} x_{j} \tag{8}$$

$$u_{ij}^{(k)} = \frac{1}{\sum_{r=1}^{c}\left(\frac{d_{ij}^{(k)}}{d_{rj}^{(k)}}\right)^{2/(m-1)}} \tag{9}$$

$$v_{i}^{(k+1)} = \frac{\sum_{j=1}^{n}\left(u_{ij}^{(k)}\right)^{m} x_{j}}{\sum_{j=1}^{n}\left(u_{ij}^{(k)}\right)^{m}} \tag{10}$$

$$L(c) = \frac{\left(\sum_{i=1}^{c}\left(\sum_{j=1}^{n} u_{ij}^{m}\right)\left\|v_{i} - \bar{x}\right\|^{2}\right)\Big/(c-1)}{\left(\sum_{i=1}^{c}\left(\sum_{j=1}^{n} u_{ij}^{m}\right)\left\|x_{j} - v_{i}\right\|^{2}\right)\Big/(n-c)} \tag{11}$$

In equation 10, the between-class and in-class intervals are presented as the numerator and denominator, respectively. A higher (c) value clearly results in more rational clustering. The ANFIS-FCM flowchart is at the Supplementary Figure S2.

Uncertainty analysis

The first step in calculating the uncertainty entails calculating the individual forecasting error (IFE) as follows:

$$IFE_j = P_j - A_j \tag{12}$$

where P_j and A_j are the jth forecasted and observed IPFP volumes, respectively. To compute the forecasting error of the entire dataset, the mean forecasting error (*MFE*) and standard deviation of the forecasting error (*SDFE*) are defined as follows:

$$MFE = \sum_{j=1}^{n} IFE_j \tag{13}$$

$$SDFE = \sqrt{\frac{\sum_{j=1}^{n} \left(IFE_j - MFE\right)^2}{n-1}} \tag{14}$$

where *n* is the number of samples. A negative (or positive) *MFE* value indicates that the prediction model underestimated (or overestimated) the actual values. The confidence band around the forecasted values may be obtained with the Wilson score approach deprived of continuity correction[15]. With this method, the 95% confidence width of uncertainty band (WUB) could be evaluated by using ±1.96 SDFE.

Performance evaluation criteria

The correlation coefficient is in the [-1, 1] range, while the other indices are in the [0, ∞] range. *SI*, *R*, *MAPE* and *RMSRE* are defined as follows:

$$SI = \frac{\sqrt{\frac{1}{n}\sum_{i=1}^{n}\left(A_i - P_i\right)^2}}{\overline{A_i}} \tag{15}$$

$$R = \left(\frac{\left(\sum_{i=1}^{n} \left(A_i - \overline{A}\right)\left(P_i - \overline{P}\right) \right)}{\sqrt{\sum_{i=1}^{n} \left(A_i - \overline{A}\right)^2 \sum_{i=1}^{n} \left(P_i - \overline{P}\right)^2}} \right) \tag{16}$$

$$MAPE = \frac{100}{n}\sum_{i=1}^{n}\left(\frac{\left|A_i - P_i\right|}{A_i}\right) \tag{17}$$

$$RMSRE = \sqrt{\frac{1}{n}\sum_{i=1}^{n}\left(\frac{A_i - P_i}{A_i}\right)^2} \quad (18)$$

where n is the sample number, A_i and P_i are the actual and predicted i^{th} samples respectively and $\overline{A}$ and $\overline{P}$ are the average actual and predicted samples, respectively.

ANFIS-FCM based pseudocode for IPFP volume prediction based on gender separation

Total cohort (Female)

```
clc
clear all
close all

%% Read data
Prompt={'BMI','Age','Adipsin/Chemerin','Adipsin/CRP'};
        Title='Enter the values of input variables';
        DefaultValues={'[23;25;27];','[56;61;45];','[3.8472;8.3602;4.2482];','[2.5135;3.5619;1.5392];'};

        PARAMS=inputdlg(Prompt,Title,3,DefaultValues);

        BMI=str2num(PARAMS{1});
        Age=str2num(PARAMS{2});
        AdipsinChemerin=str2num(PARAMS{3});
        AdipsinCRP=str2num(PARAMS{4});

        if length(Age)<2
            a1=[BMI;BMI];;
            a2=[Age;Age];;
            a3=[AdipsinChemerin;AdipsinChemerin];;
            a4=[AdipsinCRP;AdipsinCRP];;

        else
            a1=BMI;
            a2=Age;
            a3=AdipsinChemerin;
            a4=AdipsinCRP;
        end
        TestInputs=[a1 a2 a3 log(a4)];;

        z=length(Age);
        TestTargets=ones(z,1);


data.TestInputs=TestInputs;
data.TestTargets=TestTargets;

x=data.TestInputs;
t=data.TestTargets;

%% Fis generation _ random parameters
fis=genfis3(x,t,'sugeno',15);
clc

%% Replace the optimized values with initial parameters
% Inputs
fis.input(1).mf(1).params= [1.9071 24.6212];
fis.input(1).mf(2).params= [1.9849 29.9797];
fis.input(1).mf(3).params= [2.0884 31.2389];
fis.input(1).mf(4).params= [1.7651 28.0714];
fis.input(1).mf(5).params= [2.0235 29.8898];
fis.input(1).mf(6).params= [1.7475 25.6190];
fis.input(1).mf(7).params= [2.4659 33.0230];
fis.input(1).mf(8).params= [2.1805 28.5154];
fis.input(1).mf(9).params= [1.9305 28.4868];
fis.input(1).mf(10).params= [2.3424 31.7211];
fis.input(1).mf(11).params= [2.3440 32.0451];
fis.input(1).mf(12).params= [1.8776 30.0680];
fis.input(1).mf(13).params= [2.1066 29.7992];
fis.input(1).mf(14).params= [1.9102 26.7496];
fis.input(1).mf(15).params= [2.1569 27.6407];

fis.input(2).mf(1).params= [2.8850 64.3983];
fis.input(2).mf(2).params= [3.1419 60.4673];
fis.input(2).mf(3).params= [3.3879 60.9867];
fis.input(2).mf(4).params= [2.8422 62.0612];
fis.input(2).mf(5).params= [3.3942 60.1144];
fis.input(2).mf(6).params= [3.3913 62.6573];
```

```
fis.input(2).mf(7).params= [3.1878 58.4064];
fis.input(2).mf(8).params= [3.3112 58.2856];
fis.input(2).mf(9).params= [2.5721 57.9001];
fis.input(2).mf(10).params= [2.7777 59.9792];
fis.input(2).mf(11).params= [3.1307 59.5052];
fis.input(2).mf(12).params= [3.2539 61.6368];
fis.input(2).mf(13).params= [2.9698 59.6360];
fis.input(2).mf(14).params= [3.4917 56.3717];
fis.input(2).mf(15).params= [3.0059 58.0339];

fis.input(3).mf(1).params= [0.1409 1.1212];
fis.input(3).mf(2).params= [0.2330 1.2884];
fis.input(3).mf(3).params= [0.1345 1.4448];
fis.input(3).mf(4).params= [0.1874 0.8656];
fis.input(3).mf(5).params= [0.2315 1.3027];
fis.input(3).mf(6).params= [0.1665 0.9613];
fis.input(3).mf(7).params= [0.1858 1.5146];
fis.input(3).mf(8).params= [0.2155 1.0996];
fis.input(3).mf(9).params= [0.1725 1.0493];
fis.input(3).mf(10).params= [0.2615 1.3514];
fis.input(3).mf(11).params= [0.2324 1.5263];
fis.input(3).mf(12).params= [0.2559 1.5196];
fis.input(3).mf(13).params= [0.0976 1.3697];
fis.input(3).mf(14).params= [0.2652 1.0543];
fis.input(3).mf(15).params= [0.2092 1.0594];

fis.input(4).mf(1).params= [0.4813 1.3397];
fis.input(4).mf(2).params= [0.5906 0.2207];
fis.input(4).mf(3).params= [0.2938 0.5594];
fis.input(4).mf(4).params= [0.3830 1.1811];
fis.input(4).mf(5).params= [0.5833 0.1269];
fis.input(4).mf(6).params= [0.5868 0.4896];
fis.input(4).mf(7).params= [0.4583 1.2307];
fis.input(4).mf(8).params= [0.4701 1.1161];
fis.input(4).mf(9).params= [0.5690 0.3518];
fis.input(4).mf(10).params= [0.5775 0.0075];
fis.input(4).mf(11).params= [0.5796 0.3614];
fis.input(4).mf(12).params= [0.6060 0.5210];
fis.input(4).mf(13).params= [0.5094 0.8101];
fis.input(4).mf(14).params= [0.5550 0.3823];
fis.input(4).mf(15).params= [0.4703 0.8178];


% Outputs
fis.output.mf(1).params = [-826.833720492606,769.264519408740,-5851.96776095094,2141.10096623188,-12076.6100581568];
fis.output.mf(2).params = [-14908.5943098417,3854.68897596641,-68232.0271900687,-48611.9263382136,315053.167210565];
fis.output.mf(3).params = [-27522.3884813258,2336.46741876781,24669.5406673392,-184910.527198670,873897.339243090];
fis.output.mf(4).params = [-1796.75926417112,2524.89336092043,-14200.2352612096,-2381.32732784713,-89476.3586681258];
fis.output.mf(5).params = [19464.0396376293,877.488719030217,84349.6660291703,24857.3776985812,-749166.991186159];
fis.output.mf(6).params = [1557.60522033709,-204.783627372398,7943.78960047110,-2718.44040023199,-12158.0582741821];
fis.output.mf(7).params = [-473.392268408735,412.408307990210,7396.63112056444,-5572.73627569211,17818.0577177712];
fis.output.mf(8).params = [-3287.03284712200,2083.36306418919,-19331.2427665017,-7725.97418505073,71478.6375492024];
fis.output.mf(9).params = [17543.3369999959,6360.86818653105,182752.613781429,-106765.224868415,-1041137.69091593];
fis.output.mf(10).params = [1144.61907892834,-510.222394737963,-3634.81349779552,2780.17863491849,19401.4794891029];
fis.output.mf(11).params = [-1257.51136226060,135.556742003101,17763.2669273451,-4871.05982533808,24331.2310776842];
fis.output.mf(12).params = [-114.114879543919,-85.1100761226213,572.915083944015,-16.4251066990063,27416.9071444308];
fis.output.mf(13).params = [35505.8091884353,293.346964675554,354040.721716866,7509.04070831255,-1580045.35870900];
fis.output.mf(14).params = [723.212955607581,-214.756987264587,829.224937531366,-1820.53757811690,12144.4227993343];
fis.output.mf(15).params = [-2546.73162061527,6593.89334115133,19531.0610612247,-87879.3188683679,-184860.211611115];

%% FatpatVolumeMm3 calculation
TestOutputs=abs(evalfis(data.TestInputs,fis));

if length(Age)<2
   IPFPVolume =TestOutputs(1)
else
   IPFPVolume =TestOutputs
end
```

Total cohort (Male)

```
clc
clear all
close all

%% Read data
Prompt={'BMI','Age','Adipsin/Chemerin','Adipsin/CRP'};
        Title='Enter the values of input variables';
        DefaultValues={'[23;25;27]','[56;61;45]','[3.8472;8.3602;4.2482]','[2.5135;3.5619;1.5392]'};

        PARAMS=inputdlg(Prompt,Title,3,DefaultValues);

        BMI=str2num(PARAMS{1});
        Age=str2num(PARAMS{2});
        AdipsinChemerin=str2num(PARAMS{3});
        AdipsinCRP=str2num(PARAMS{4});

        if length(Age)<2
            a1=[BMI;BMI];
            a2=[Age;Age];
            a3=[AdipsinChemerin;AdipsinChemerin];
            a4=[AdipsinCRP;AdipsinCRP];
```

```matlab
        else
            a1=BMI;
            a2=Age;
            a3=AdipsinChemerin;
            a4=AdipsinCRP;
        end
        TestInputs=[a1 a2 a3 log(a4)];

        z=length(Age);
        TestTargets=ones(z,1);



data.TestInputs=TestInputs;
data.TestTargets=TestTargets;

x=data.TestInputs;
t=data.TestTargets;
%% FIS generation _ random parameters
fis=genfis3(x,t,'sugeno',21);
clc
%% Replace the optimized values with initial parameters
% Inputs
fis.input(1).mf(1).params= [1.2652 28.7322];
fis.input(1).mf(2).params= [1.6414 26.9765];
fis.input(1).mf(3).params= [1.4436 32.5489];
fis.input(1).mf(4).params= [1.0043 29.9588];
fis.input(1).mf(5).params= [1.1273 28.9431];
fis.input(1).mf(6).params= [0.9533 30.8697];
fis.input(1).mf(7).params= [1.0629 29.1628];
fis.input(1).mf(8).params= [1.3517 30.1294];
fis.input(1).mf(9).params= [1.2812 29.9512];
fis.input(1).mf(10).params= [1.7854 32.5970];
fis.input(1).mf(11).params= [1.5018 30.0320];
fis.input(1).mf(12).params= [1.3606 31.4711];
fis.input(1).mf(13).params= [1.1152 30.0370];
fis.input(1).mf(14).params= [1.2189 28.1825];
fis.input(1).mf(15).params= [1.2515 31.0093];
fis.input(1).mf(16).params= [1.6540 25.3408];
fis.input(1).mf(17).params= [1.2892 29.6405];
fis.input(1).mf(18).params= [1.5687 32.8197];
fis.input(1).mf(19).params= [1.3573 29.1060];
fis.input(1).mf(20).params= [1.0125 29.1702];
fis.input(1).mf(21).params= [1.2763 30.3657];

fis.input(2).mf(1).params= [3.1416 61.1130];
fis.input(2).mf(2).params= [3.1035 58.7476];
fis.input(2).mf(3).params= [2.8981 58.7386];
fis.input(2).mf(4).params= [3.5124 56.1116];
fis.input(2).mf(5).params= [3.0726 62.7039];
fis.input(2).mf(6).params= [3.3647 63.6571];
fis.input(2).mf(7).params= [3.5957 65.6807];
fis.input(2).mf(8).params= [3.1526 59.6038];
fis.input(2).mf(9).params= [3.1612 58.8522];
fis.input(2).mf(10).params= [2.9262 59.4751];
fis.input(2).mf(11).params= [3.1406 59.3489];
fis.input(2).mf(12).params= [3.0385 57.3297];
fis.input(2).mf(13).params= [3.0357 59.3322];
fis.input(2).mf(14).params= [3.1715 61.5692];
fis.input(2).mf(15).params= [3.1442 63.8223];
fis.input(2).mf(16).params= [3.2478 61.1142];
fis.input(2).mf(17).params= [2.9990 62.5093];
fis.input(2).mf(18).params= [3.2330 64.3659];
fis.input(2).mf(19).params= [4.0946 68.2012];
fis.input(2).mf(20).params= [3.0954 61.5533];
fis.input(2).mf(21).params= [2.8776 59.4709];

fis.input(3).mf(1).params= [0.2111 1.4039];
fis.input(3).mf(2).params= [0.1681 1.0925];
fis.input(3).mf(3).params= [0.1540 1.4052];
fis.input(3).mf(4).params= [0.3945 1.9902];
fis.input(3).mf(5).params= [0.9811 3.7262];
fis.input(3).mf(6).params= [0.6187 2.0882];
fis.input(3).mf(7).params= [0.1489 1.3484];
fis.input(3).mf(8).params= [0.2421 1.3424];
fis.input(3).mf(9).params= [0.1729 1.1440];
fis.input(3).mf(10).params= [0.2395 1.4429];
fis.input(3).mf(11).params= [0.1493 1.3316];
fis.input(3).mf(12).params= [0.2102 0.9687];
fis.input(3).mf(13).params= [0.1103 1.0939];
fis.input(3).mf(14).params= [0.2238 1.5032];
fis.input(3).mf(15).params= [0.1408 1.2693];
fis.input(3).mf(16).params= [0.1493 1.3779];
fis.input(3).mf(17).params= [0.1563 1.1299];
fis.input(3).mf(18).params= [0.3531 1.0572];
fis.input(3).mf(19).params= [0.1320 1.3793];
fis.input(3).mf(20).params= [0.3675 1.8265];
fis.input(3).mf(21).params= [0.2040 1.4065];

fis.input(4).mf(1).params= [0.3048 1.8305];
fis.input(4).mf(2).params= [0.3534 1.3530];
fis.input(4).mf(3).params= [0.3196 1.4879];
fis.input(4).mf(4).params= [0.1897 0.7404];
fis.input(4).mf(5).params= [0.3901 2.1298];
```

```
fis.input(4).mf(6).params= [0.2292 1.8005];
fis.input(4).mf(7).params= [0.4737 1.2237];
fis.input(4).mf(8).params= [0.3400 1.6341];
fis.input(4).mf(9).params= [0.3280 1.4959];
fis.input(4).mf(10).params= [0.3374 1.5230];
fis.input(4).mf(11).params= [0.3721 1.4551];
fis.input(4).mf(12).params= [0.3434 1.5138];
fis.input(4).mf(13).params= [0.4561 0.7406];
fis.input(4).mf(14).params= [0.3254 1.8034];
fis.input(4).mf(15).params= [0.4730 0.9677];
fis.input(4).mf(16).params= [0.2239 2.0033];
fis.input(4).mf(17).params= [0.5377 0.6068];
fis.input(4).mf(18).params= [0.2039 1.7421];
fis.input(4).mf(19).params= [0.4776 1.2974];
fis.input(4).mf(20).params= [0.4667 1.2283];
fis.input(4).mf(21).params= [0.3234 1.7683];

% Outputs
fis.output.mf(1).params = [-89256.1327089724,8897.92815956006,718470.564358239,193860.564863406,784476.577953986];
fis.output.mf(2).params = [499.010096827655,-534.173961330704,-9139.88195556069,-1757.06376470105,55689.4244765867];
fis.output.mf(3).params = [-189.714839497532,-2137.18934622544,73543.2373651745,50424.6611137503,-15535.9904449384];
fis.output.mf(4).params = [5229.51403913427,-818.589926940509,-1397.73389356727,29349.3836174541,-102300.187771111];
fis.output.mf(5).params = [-247.454995778685,-137.744345847725,66.0501254306017,-187.456840206216,44893.6910844611];
fis.output.mf(6).params = [1562.02979945106,1230.73036768832,18369.9225572958,10287.1315096775,-166502.753426449];
fis.output.mf(7).params = [2094.37527521318,8404.77481809602,92947.2695807591,24777.9762372340,-763312.731045743];
fis.output.mf(8).params = [-212494.712255855,86788.1559383042,866361.105612832,599669.754173434,-980706.877913351];
fis.output.mf(9).params = [60823.4194365018,-5066.97024477580,-474984.706739506,266235.113420192,-1213456.68026691];
fis.output.mf(10).params = [-183.894799908000,75.5900042660704,-312.584919387115,-3339.56335708479,44937.6921021419];
fis.output.mf(11).params = [11032.0764383067,-15280.4878706398,-298372.663179735,15496.2225543327,1199443.04383223];
fis.output.mf(12).params = [-2500.18184266817,1639.39484211338,-10401.4128223203,6860.28415673561,38167.0230894190];
fis.output.mf(13).params = [-7727.85680337128,-723.758181337889,-57838.1428711436,27710.5954990440,353711.587586557];
fis.output.mf(14).params = [-25217.1400482926,5231.67406300640,82148.0901117922,14543.3890866292,51179.5728007187];
fis.output.mf(15).params = [-6850.48315223950,349.711635957004,23583.2048066623,-6846.48334738437,217177.338049463];
fis.output.mf(16).params = [-2931.53377597877,48.7284339697663,-2313.80572175287,-5340.24380263931,109407.390966829];
fis.output.mf(17).params = [462.406293672301,-65.4119679874121,10021.5003536354,1778.66101603952,15810.4350522424];
fis.output.mf(18).params = [-555.941948138538,44.3952717011562,-5599.49318425191,3651.53670154622,45885.3403885497];
fis.output.mf(19).params = [-383.215215737109,495.523783867351,-677.048960968381,182.113660160332,-520.875399081657];
fis.output.mf(20).params = [-260.923735488841,-263.317084731361,14726.4952354215,3192.36133910797,23623.1861419690];
fis.output.mf(21).params = [162004.904385953,-58943.5247201858,-260472.380892254,-473722.003626041,-229396.190631499];

%% FatpatVolumeMm3 calculation
TestOutputs=abs(evalfis(data.TestInputs,fis));

if length(Age)<2
   IPFPVolume=TestOutputs(1)
else
   IPFPVolume=TestOutputs
end
```

REFERENCES

1 Martel-Pelletier, J. *et al.* The ratio adipsin/MCP-1 is strongly associated with structural changes and CRP/MCP-1 with symptoms in obese knee osteoarthritis subjects: data from the Osteoarthritis Initiative. *Osteoarthritis Cartilage* **28**, 1163-1173 doi:10.1016/j.joca.2019.04.016 (2019).

2 Dice, L. Measures of the amount of ecologic association between species. *Ecology* **26**, 297-302 (1945).

3 Wang, K. *et al.* Serum levels of interleukin-17 and adiponectin are associated with infrapatellar fat pad volume and signal intensity alteration in patients with knee osteoarthritis. *Arthritis Res Ther* **18**, 193, doi:10.1186/s13075-016-1088-9 (2016).

4 Eberhart, R. C. & Kennedy, J. in *MHS'95, Proceedings of the sixth international symposium on micro machine and human science.* 39-43 (IEEE).

5 Van Den Bergh, F. *An analysis of particle swarm optimizers* PhD thesis, University of Pretoria, (2001).

6 Qasem, S. N., Ebtehaj, I. & Bonakdari, H. Potential of radial basis function network with particle swarm optimization for prediction of sediment transport at the limit of deposition in a clean pipe. *Sustain Water Resour Manag* **3**, 391-401, doi:https://doi.org/10.1007/s40899-017-0104-9 (2017).

7 Gholami, A. *et al.* Uncertainty analysis of intelligent model of hybrid genetic algorithm and particle swarm optimization with ANFIS to predict threshold bank profile shape based on digital laser approach sensing. *Measurement* **121**, 294-303 (2018).

8 Gharabaghi B., Bonakdari H., Ebtehaj I. Hybrid Evolutionary Algorithm Based on PSOGA for ANFIS Designing in Prediction of No-Deposition Bed Load Sediment Transport in Sewer Pipe. In: Arai K., Kapoor S., Bhatia R. (eds) Intelligent Computing. SAI 2018. *Advances in Intelligent Systems and Computing*, **vol 857**. Springer, New York, USA. https:/doi.org/10.1007/978-3-030-01177-2_8 (2019).

9 Ebtehaj, I., Bonakdari, H. & Zaji, A. H. An expert system with radial basis function neural network based on decision trees for predicting sediment transport in sewers. *Water Sci Technol* **74**, 176-183, doi:10.2166/wst.2016.174 (2016).

10 Ebtehaj, I. & Bonakdari, H. Bed load sediment transport estimation in a clean pipe using multilayer perceptron with different training algorithms. *KSCE Journal of Civil Engineering* **20**, 581-589, doi:10.1007/s12205-015-0630-7 (2016).

11 Bonakdari, H., Moeeni, H., Ebtehaj, I. & al., e. New insights into soil temperature time series modeling: linear or nonlinear? *Theor Appl Climat* **135**, 1157-1177 (2019).

12 Ebtehaj, I., Bonakdari, H. & Sharifi, A. Design criteria for sediment transport in sewers based on self-cleansing concept. *J Zhejiang Univ-SC A* **15**, 914-924 (2014).

13 Jang, J. S. R. ANFIS: Adaptive-Network-based Fuzzy Inference System. *IEEE T Syst Man Cyb* **23**, 665-685. https://doi.org/610.1109/1121.256541 (1993).

14 Ebtehaj, I. & Bonakdari, H. Performance Evaluation of Adaptive Neural Fuzzy Inference System for Sediment Transport in Sewers. *Water Resources Management* **28**, 4765-4779, doi:10.1007/s11269-014-0774-0 (2014).

15 Ebtehaj, I., Bonakdari, H. & Gharabaghi, B. A reliable linear method for modeling lake level fluctuations. *Journal of Hydrology* **570**, 236-250, doi:https://doi.org/10.1016/j.jhydrol.2019.01.010 (2019).
