# Supplementary material for: Serum adipokines/related inflammatory factors and ratios as predictors of infrapatellar fat pad volume in osteoarthritis: Applying comprehensive machine learning approaches
Source: Sci Rep. 2020 Jun 19;10:9993. doi: 10.1038/s41598-020-66330-0 (PMC7305166; doi:10.1038/s41598-020-66330-0)
Supplement: Supplementary file 3 — Supplementary information 3. [file 41598_2020_66330_MOESM3_ESM.pdf]

Serum adipokines/related inflammatory factors and ratios as predictors of infrapatellar fat pad volume in osteoarthritis: Applying comprehensive machine learning approaches

Hossein Bonakdari, PhD, Ginette Tardif, PhD, François Abram, PhD, Jean-Pierre Pelletier, MD, Johanne Martel-Pelletier, PhD

SUPPLEMENTARY FIGURES

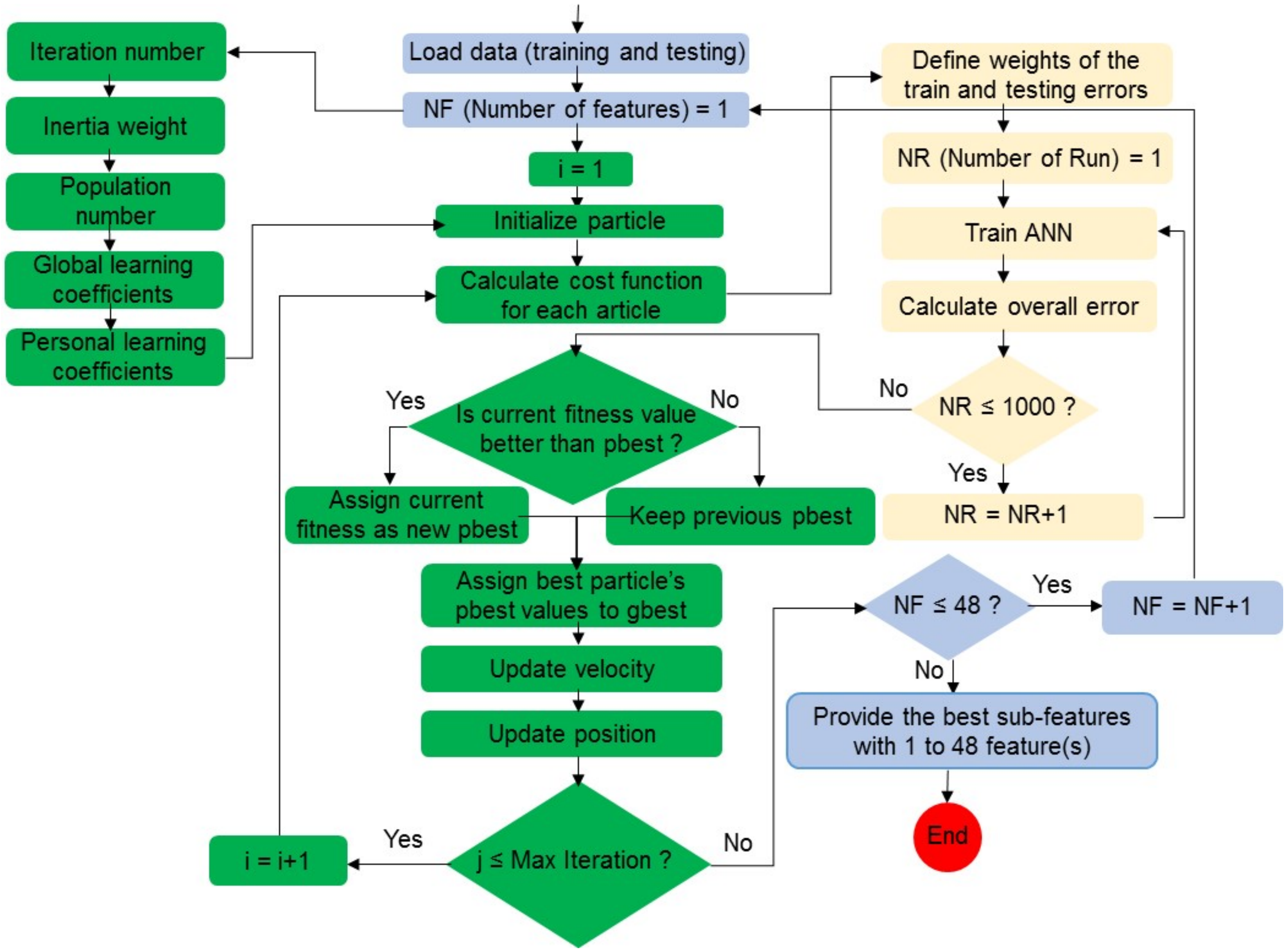

Figure S1. Flow chart of the proposed particle swarm optimization (PSO)-based feature selection method
ANN, Artificial neural network.

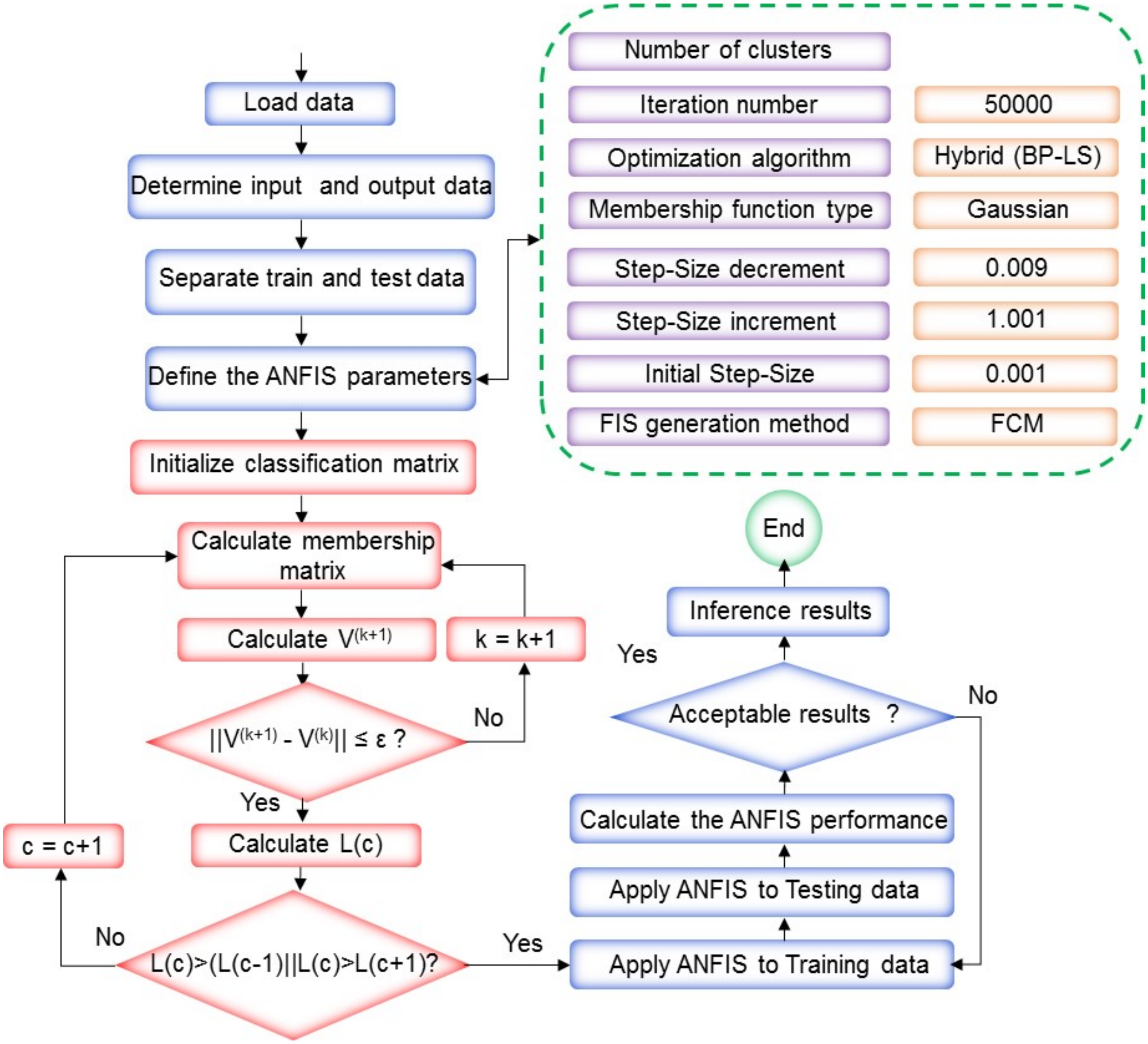

Figure S2. Adaptive neuro-fuzzy inference system (ANFIS) embedded with fuzzy c-means (FCM) clustering (ANFIS-FCM) flowchart.

BP-LS, backpropagation and least square; FCM, fuzzy c-means; FIS, fuzzy inference system; $V^{(k)}$, the clustering center matrix; ε threshold value; L(c), adaptive function of clustering; c and k numerator.

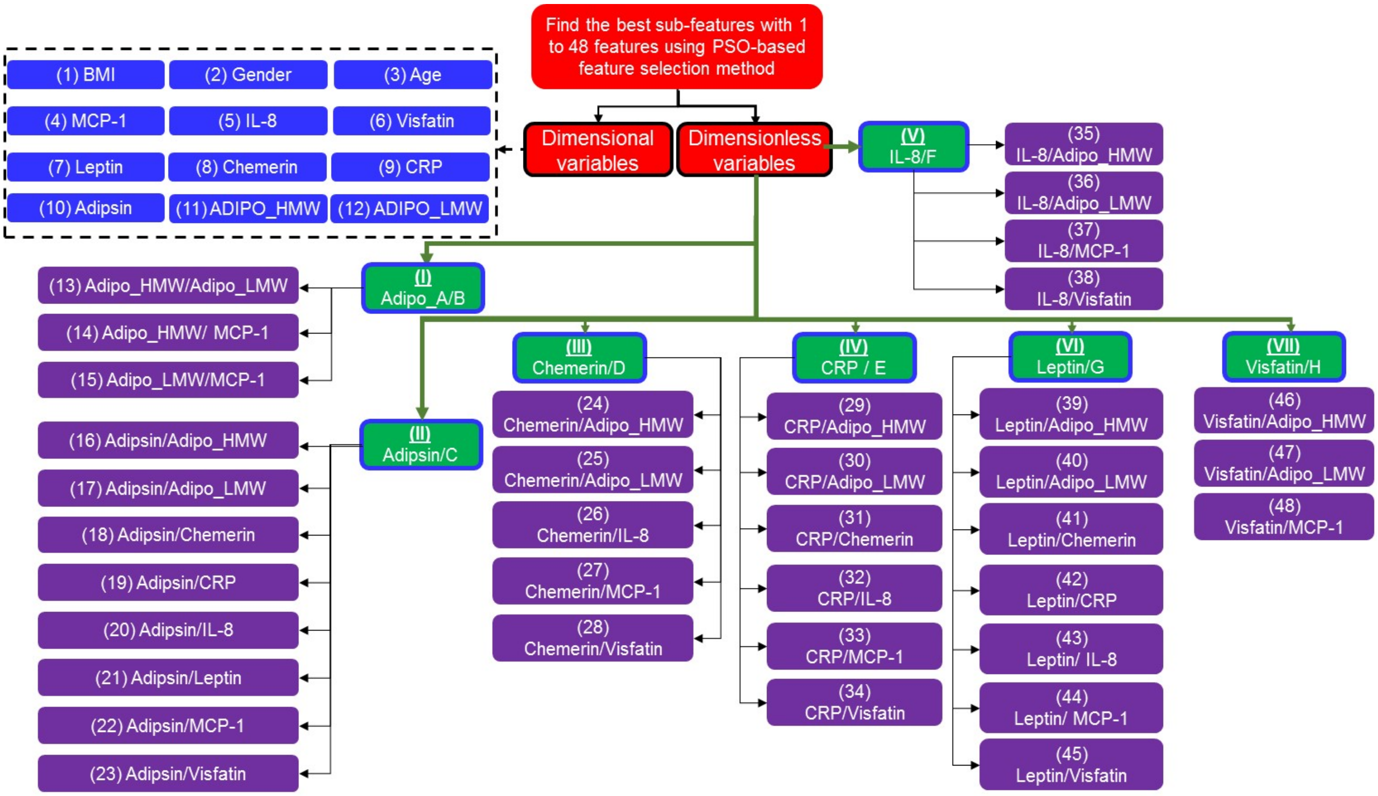

Figure S3. Definition of the 48 dimensional features and their ratios to find the best sub-features of models with 1 to 48 features. Numbers (1)-(3), risk factor variables; (4)-(12), biomarkers values; (13)-(15), Adipo_HMW and Adipo_LMW ratios (I); (16)-(23), Adipsin ratios (II); (24)-(28), Chemerin ratios (III); (29)-(34), CRP ratios (IV); (35)-(38), IL-8 ratios (V); (39)-(45), Leptin ratios (VI); (46)-(48), Visfatin ratios (VII).
Adipo_HMW, Adiponectin high molecular weight; Adipo_LMW, adiponectin low molecular weight; BMI, body mass index; CRP, C-reactive protein; IL-8, interleukin 8 (pg/ml); MCP-1, monocyte chemoattractant protein-1; PSO, particle swarm optimization.

Figure S4. Total Cohort

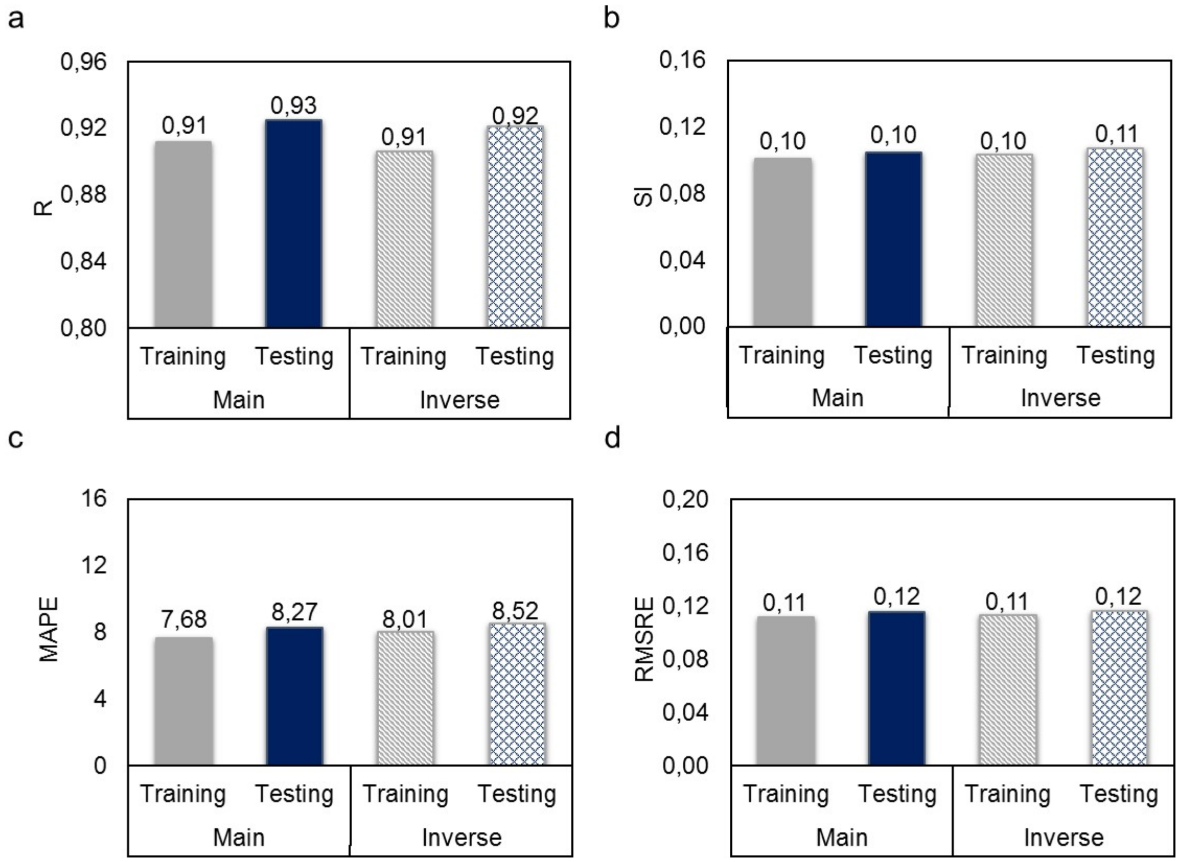

Figure S4. High-BMI

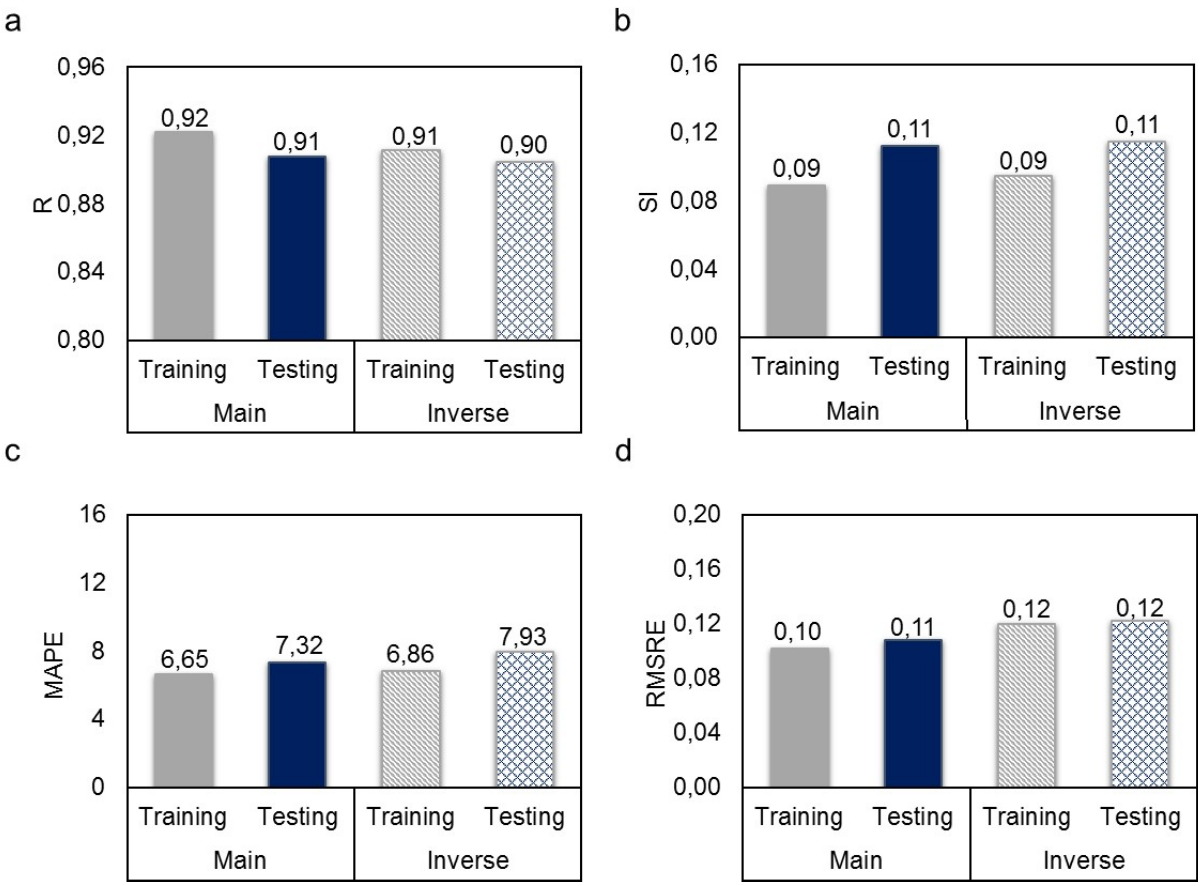

Figure S4. Low-BMI

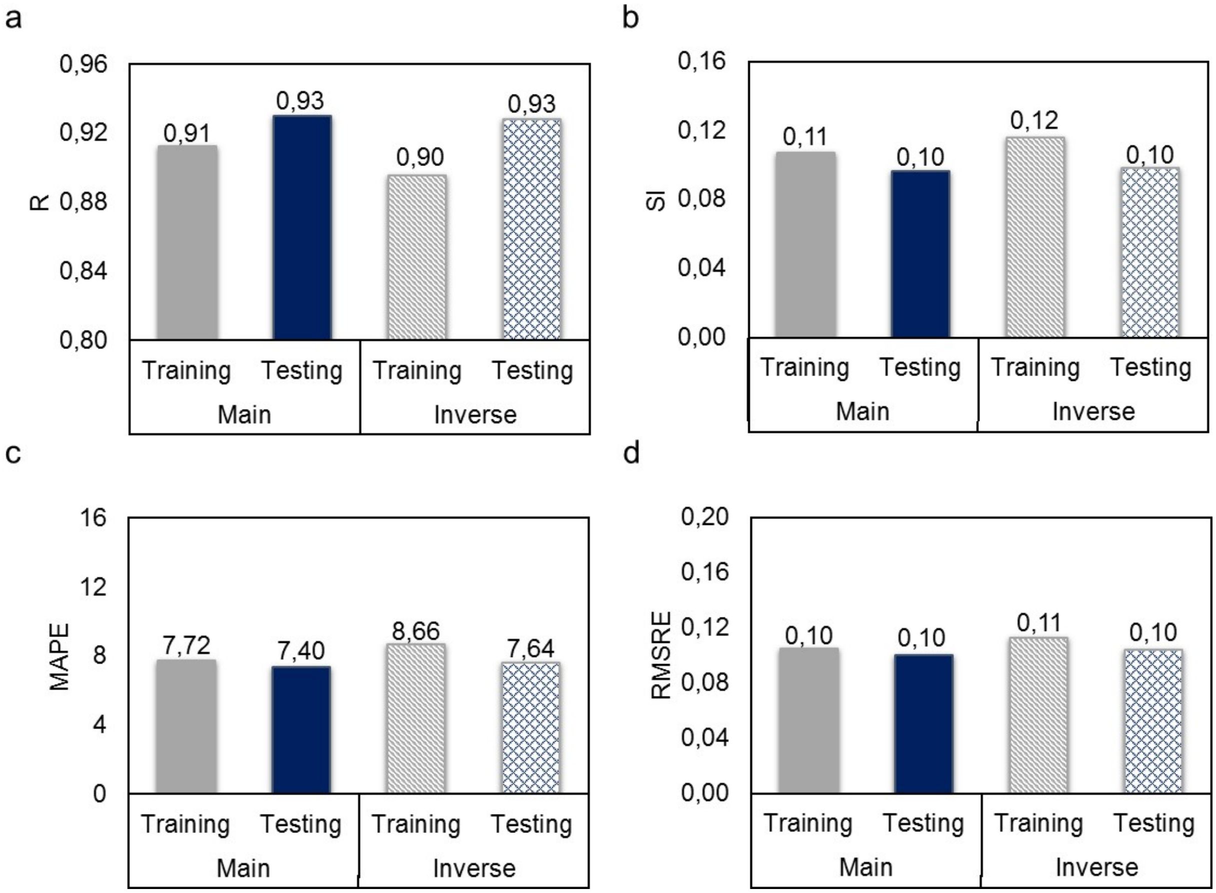

Performance evaluation of the adaptive neuro-fuzzy inference system embedded with fuzzy c-means clustering (ANFIS-FCM) based model in infra patellar fat pad (IPFP) volume prediction in comparison with the inverse of the input features for training and testing stages for Total Cohort, High-bone mass index (BMI) and Low-BMI. Main refers to the ratios as expressed in Supplementary Figure S3; inverse is the inverse ratio.
a) R, coefficient correlation; b) SI, scatter index; c) MAPE, mean absolute percentage error; d) RSMRE, root mean square relative error.
